# 3D Expansion–PALM (PhotoActivated Localization Microscopy) Dissects Protein–Protein Interactions Down to the Molecular Scale in Bacteria

**DOI:** 10.3390/microorganisms14040772

**Published:** 2026-03-28

**Authors:** Chiara Caldini, Sara Del Duca, Alberto Vassallo, Giulia Semenzato, Renato Fani, Francesco Saverio Pavone, Lucia Gardini

**Affiliations:** 1 European Laboratory for Non-Linear Spectroscopy (LENS), Via Nello Carrara 1, 50019 Sesto Fiorentino, Italy; francesco.pavone@unifi.it; 2 Department of Physics and Astronomy, University of Florence, Via Giovanni Sansone 1, 50019 Sesto Fiorentino, Italy; 3 Department of Biology, University of Florence, Via Madonna del Piano 6, 50019 Sesto Fiorentino, Italy; sara.delduca@crea.gov.it (S.D.D.); alberto.vassallo@unicam.it (A.V.); giuliasemenzato@cnr.it (G.S.); renato.fani@unifi.it (R.F.); 4 Research Centre for Genomics and Bioinformatics, Council for Agricultural Research and Economics (CREA), Via S. Protaso 69, 29017 Fiorenzuola d’Arda, Italy; 5 Institute for Sustainable Plant Protection, National Research Council of Italy, 50019 Sesto Fiorentino, Italy; 6 National Institute of Optics, National Research Council (CNR-INO), Largo Enrico Fermi 6, 50125 Florence, Italy; 7 Institute for Complex Systems, National Research Council, Via Madonna del Piano 10, 50019 Sesto Fiorentino, Italy

**Keywords:** *E. coli*, super-resolution microscopy, expansion microscopy, expansion–PALM, dual color, molecular distance

## Abstract

Super-resolution microscopy has transformed biological imaging by enabling nanoscale visualization of cellular structures beyond the diffraction limit. However, its effective application in highly dense molecular environments still poses challenges. This is the case for 3D PhotoActivated Localization Microscopy (PALM) achieved through astigmatism in bacterial cells. The limited volume of a single bacterium highly increases the probability of the intensity profiles emitted by single chromophores to overlap, thus strongly decreasing the number of localizations, leading to dramatic undersampling. Dual-color 3D super-resolution in *Escherichia coli* is achieved through a combination of PALM with Expansion Microscopy (Ex-PALM). PALM provides high specificity through photoactivable (PA) fusion proteins and high localization precision, while ExM physically expands the specimen and separate densely packed molecules. This hybrid approach enables dual-color 3D single-molecule localization with about 3 nm spatial resolution, thus allowing one to measure distances down to the molecular scale. This is achieved by optimizing ExM protocols in bacteria to achieve a 4-fold isotropic expansion, by minimizing both chromatic aberrations and signal crosstalk, and by improving single-molecule sensitivity through highly selective inclined illumination. The method is applied to measure the spatial distribution of HisF and HisH proteins, involved in *E. coli* histidine biosynthesis. By tagging each protein with a photoactivable fluorescent protein, Ex-PALM reveals that after being synthetized, they co-localize in the bacterial volume with an average 3D distance of 19 nm. By combining labeling specificity with Ex-PALM, an effective method is developed for studying molecular organization in prokaryotes and in high-density samples in general, such as cell organelles or molecular condensates, with broad applications in microbiology, synthetic biology, and cellular biophysics.

## 1. Introduction

Bacteria represent particularly challenging samples for fluorescence microscopy due to their extremely limited cellular volume. Single-molecule localization imaging has proven effective in revealing novel structural and functional details of intracellular organization in both eukaryotic and bacterial cells [1,2,3,4,5,6]. However, the application of 3D single-molecule imaging based on astigmatism—the most widely used approach for axial localization—strongly depends on both the density of the protein of interest within the bacterial cell and the photoswitching properties of the selected fluorophore. In highly dense molecular environments, overlapping elliptic intensity profiles can become a predominant factor, leading to reduced image resolution and severe undersampling. This effect is further exacerbated in co-localization experiments, where the photoswitching properties of different fluorophores must be matched, thereby undermining the applicability of multicolor 3D super-resolution microscopy in bacteria.

Expansion Microscopy (ExM) is an imaging technique that physically magnifies biological samples by embedding them in a swellable polyelectrolyte gel, achieving approximately 4-fold magnification. This enables high-resolution imaging with diffraction-limited microscopes, such as confocal microscopes, reaching a resolution of about 70 nm. Since its introduction in 2015 [7], ExM has had a profound impact on biology and biophysics by giving access to the ultrastructures of cells and tissues, enabling new discoveries in areas such as cytoskeletal organization [8,9,10], synaptic connectivity [11,12], and RNA spatial organization [13,14]. During the years, the combination of ExM with other super-resolution microscopy (SRM) techniques (including SIM, STED, STORM, etc.) has been explored to reach nanometer-scale image resolution [15,16,17,18,19]. Currently, Ex-SRM has been mainly applied to the study of structures within eukaryotic cells, where its enhanced resolution has provided new insights into complex systems like the cytoskeleton, nuclear pores, and other subcellular structures [20,21,22].

Ex-SRM is even more challenging in prokaryotic cells, such as bacteria, due to the rigid cell wall, which hampers isotropic sample expansion and blocks label penetration, as well as the crowded, charged cytosol, which interferes with specific labeling and leads to difficulties in target detection and potential imaging artifacts. Consequently, to date, only a few studies have applied ExM–SRM to microbiology [23].

In this work, a methodology is presented to combine ExM with Photoactivated Localization Microscopy (PALM) [24] in bacteria, enabling the extraction of quantitative data on protein spatial distribution. PALM microscopy makes use of fluorescent proteins (FPs) genetically fused to the target for labeling. This approach offers several advantages for combination with ExM: (i) direct anchoring to hydrogel using functionalized crosslinkers like Acryloyl-X or NHS-ester derivatives (such as Methacrylic Acid N-HydroxySuccinimide ester, shortly MA-NHS), ensuring high retention and spatial accuracy after expansion, (ii) reduced labeling footprint, as FPs are smaller than antibody complexes and do not introduce significant steric hindrance, making them ideal for labeling densely packed proteins or nanoscale structures, (iii) uniform, highly efficient, and specific labeling across the entire sample, and (iv) no hydrogel shrinking, as PALM does not require switching buffers.

Specifically, in this study, PALM imaging was combined with a protein-retention ExM (proExM) protocol [25] to unveil bacterial subcellular organization. Contrary to the historical perception of bacterial cells as disorganized “bags of enzymes”, over the years, several studies revealed that the cytoplasm of prokaryotes contains various highly ordered structures, such as the nucleoid, multienzyme complexes, metabolons, etc., all of which play a role in maintaining spatial organization [26,27,28,29]. Among these, a specific form of organization within prokaryotes is the metabolon, a complex of sequential enzymes and/or stable multienzyme assemblies that transiently associate to catalyze consecutive metabolic reactions. Metabolons are crucial for channeling metabolic pathways, thus enabling the preferential transfer of an intermediate from one enzyme to a physically adjacent one and restricting diffusion into the surrounding environment. This process offers protection for unstable or scarce metabolites by keeping them in a protein-bound state and provides a metabolic advantage through the maintenance of concentration gradients, thus yielding kinetic advantages [27]. Among the different metabolic pathway, the study of the histidine biosynthetic pathway, begun over 50 years ago [30], has been pivotal in revealing fundamental biology mechanisms, such as amino acid biosynthesis, enzyme function, and metabolic regulation, and it represents a cornerstone in modern cell biology [31,32,33,34,35,36]. Previous molecular biology studies on *his* gene fusions in different phylogenetic groups suggested enzyme compartmentalization but lacked spatial evidence [33,37,38]. To study bacterial subcellular structures, an imaging approach is required. To demonstrate the molecular compartmentalization of histidine biosynthetic enzymes, the spatial distribution of HisH-HisF proteins in *E. coli* was imaged, as these proteins are known to interact based on *BACTH system* results [39]. To do this, the *E. coli* strain FB1, harboring a complete deletion of the histidine biosynthetic operon (in order to avoid any interference of the chromosomally encoded HisH and HisF proteins), was transformed with a single recombinant plasmid expressing HisH and HisF proteins, each fused to photoactivable fluorescent proteins: PAmCherry1 and PAGFP, respectively (see Supplementary Figure S1, top). On the other hand, the same *E. coli* strain was transformed with a recombinant plasmid carrying a pair of non-interacting proteins, HisH and apramycin acetyl transferase (encoded by the *aac(3)IV* gene), fused to PAmCherry1 and PAGFP, respectively, and used as a negative control (see Supplementary Figure S1, bottom). PAmCherry1 and PAGFP were chosen for their similar photoswitching properties and spectral separation, making them ideal for simultaneous multicolor imaging.

To achieve 3D co-localization of PAmCherry1 and PAGFP in both strains, we optimized 3D multicolor PALM. First, the sample was excited with an inclined beam to increase the signal-to-background ratio and improve the single-molecule sensitivity in the thick expanded gel. Fluorescence crosstalk and chromatic aberrations were then finely corrected, as they affect single-molecule localization accuracy and hinder measuring distances down to the molecular scale. The performance of these optimizations was first assessed using 2D PALM on both expanded and non-expanded bacteria, where we achieved a mean single-molecule localization accuracy of 5.8 nm and 6.7 nm for PAmCherry1 and PAGFP, respectively (non-expanded) and of 1.6 nm and 1.7 nm (expanded bacteria) for PAmCherry1 and PAGFP, respectively. An analysis method was then established to quantify co-localization between single molecules, enabling the measurement of intermolecular distances down to 12 nm in non-expanded bacteria and to 6.8 nm in expanded bacteria in 2D. Finally, the proExM protocol was optimized to achieve a 4-fold isotropic expansion of bacteria, enabling 3D single-molecule co-localization, which was otherwise impaired by the limited bacterial volume with respect to the wide spatial distribution of the astigmatic PSF out of the focal plane. A mean 3D localization accuracy of 2.7 nm and 3.2 nm (for PAmCherry1 and PAGFP, respectively) was achieved, allowing for the measurement of a 3D intermolecular distance of 19 nm between co-localizing proteins, consistent with proteins steric hindrances.

## 2. Materials and Methods

### 2.1. Design of Recombinant Plasmids

The two plasmids used in this work were derived from the pSEVA224 expression vector, which has the IPTG-inducible promoter P_trc_ and the kanamycin resistance gene (*neo*). The inserts carrying the two fusion genes (i.e., for the positive control, the PHPF insert carrying PAmCherry1-*hisH* and PAGFP-*hisF*, and for the negative control, the PHPA insert carrying PAmCherry1-*hisH* and PAGFP-*aac(3)IV;* see Supplementary Figure S1) were synthesized by the external company Bio-Fab Research s.r.l. (Rome, Italy) and cloned between the *Eco*RI and *Bam*HI restriction sites of pSEVA224. The two fusion genes of each insert are under the control of the same promoter P_trc_ but are translated independently because of distinct upstream ribosome binding sites (RBSs). Inserts were also designed to include three glycine residues between the two portions of each fusion protein: this element was added as a hinge region to ensure the correct folding and prevent steric hindrance between the two portions of the fusion proteins. Upon construction, plasmids were used to transform *E. coli* FB1 competent cells: this specific strain was used because it lacks the entire *his* operon, thus avoiding the co-presence of fluorescent-tagged proteins (i.e., PAmCherry1-HisH and PAGFP-HisF) and wild-type ones (i.e., HisH and HisF).

### 2.2. Multicolor PALM Imaging

The experimental setup (Supplementary Figure S2) features a Nikon ECLIPSE TE300 (Nikon, Tokyo, Japan) inverted fluorescence microscope equipped with three diode lasers (552, 488, and 405 nm) for excitation and an Electron-Multiplying CCD (EMCCD) camera for detection (Andor iXon X3, Oxford Instruments, Abingdon, UK). The lasers are combined via dichroic mirrors and focused through a 60× oil immersion TIRF objective (1.49 NA). Motorized translators control the angle of incidence, allowing for different illumination modes (epifluorescence or HILO). In HILO mode, the beam thickness can be adjusted to 3 μm using a linear slit (as shown in Supplementary Figure S2), thus improving the signal-to-noise ratio by a factor of 2 compared to full-field inclined illumination [3,40]. In this case, the excitation lasers were configured with an inclination of 69° inside the sample and a beam thickness of 3.8 μm. Emitted fluorescence is collected, filtered, and projected onto the EMCCD camera with 3× magnification, yielding an 82 nm pixel size over a 42 × 42 μm^2^ field of view. Two-color imaging is achieved by splitting the emissions of the two fluorophores with dichroic mirrors and filters, while 3D imaging is enabled by a cylindrical lens introducing astigmatism for z-position encoding [41]. The sample and objective are mounted on piezoelectric stages for precise xyz positioning. A custom LabVIEW program controls the system, with active stabilization for long-term 3D single-molecule experiments [42].

Simultaneous multicolor acquisition was performed with 80 ms exposure time and 400 EMCCD Gain. First, the 488 nm laser was activated at about 200 W/cm^2^ to excite PAGFP, acquiring 10–20 initial frames to assess and correct the crosstalk. Then, the 405 nm and 552 nm lasers were activated, respectively, at about 5 mW/cm^2^ and 500 W/cm^2^, to excite PAmCherry1, initiating simultaneous imaging. Each acquisition lasted about 160 s, ensuring PAGFP was fully photobleached.

For multicolor PALM on non-expanded bacteria, a sample with fixed bacteria embedded in agarose gel was assembled as described in Supplementary Protocols and Supplementary Figure S3.

### 2.3. Chromatic Aberration Correction

Chromatic aberrations affect multicolor imaging by causing different wavelengths to travel distinct optical paths and thus be focused on different focal planes. While achromatic doublets can minimize these aberrations, finer corrections are required to achieve nanometer-level precision in multicolor super-resolution imaging. In 2005, Churchman et al. developed a technique called Single-Molecule High-Resolution Co-localization (SHREC) [43] that, by using a non-rigid transformation function to correct for lateral chromatic aberration, can measure intermolecular distances with 10 nm accuracy. In this work, a custom Matlab 2025b algorithm was developed that corrects both lateral and axial chromatic aberrations by combining the lateral correction of the SHREC technique with an axial focal shift correction between different channels. This enables measuring intermolecular distances in 3D with nanometer precision. The algorithm requires a calibration step by using fiducial markers emitting fluorescence in both acquisition channels to estimate the mapping function. To ensure accurate chromatic aberration correction, before each set of measurements, a calibration sample with multi-emission fluorescent beads attached to the glass coverslip was scanned through the entire field of view in 600 nm steps (see Supplementary Protocols) using a piezoelectric stage with a positioning error of 1 nm.

**Figure 1:**
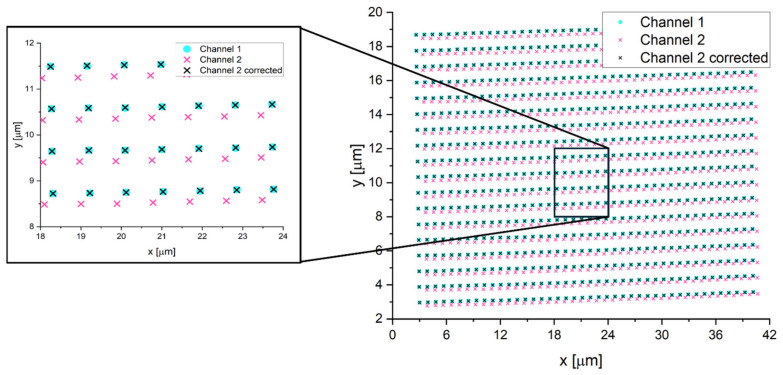
Example of an acquisition where localizations in channel 2 are mapped in channel 1 using the non-rigid transformation function obtained with the proposed algorithm. Scanning step size: 900 nm.

The algorithm uses the Matlab fitgeotform2d function to fit a local weighted mean (LWM) transformation to control point pairs from the two channels (Supplementary Figure S4). Given two sets of points, two functions are calculated: f(x,y) and g(x,y), one for each coordinate. To calculate these transformations, the algorithm selects control points, measures distances to the eight nearest neighbors, and defines a radius of influence for the transformation at each point. It then infers a polynomial transformation function for each control point using neighboring points. These functions, along with their radii of influence, form the basis for mapping each point in one channel to the corresponding point in the other channel. The global transformation function at an arbitrary point is computed as a weighted sum of local polynomials, where weights depend on the distance from the control point and ensure that polynomials influence only within their radius of influence (Supplementary Figure S4). This weighting procedure also guarantees smooth and continuous transformations across all points. The transformation functions allow for mapping between corresponding coordinates in different channels. Thus, for an objected emitting in both channels (here referred to as a “control point”), by using the localized position of the object in the magenta channel, the algorithm calculates its position in the cyan channel. This method finely corrects for lateral chromatic aberration, as shown in Figure 1, where grids of points from two channels were corrected using this algorithm.

To estimate an accuracy for this registration procedure, the Fiducial Registration Error (FRE) is calculated. The FRE measures the distance between corresponding control points in the two channels after the transformation. For N control points, the FRE along the x-axis between control points in channel one ($x_{\left(i,1\right)}$ with i = 1, …, N) and corresponding control points in channel two obtained through the transformation function ($f_{LWM}\left(x_{\left(i,2\right)}\right)$ with i = 1, …, N) is calculated as:

(1) $${FRE}_{x}=\sqrt{\frac{1}{N}\sum_{i=1}^{N}{\left[x_{\left(i,1\right)}-f_{LWM}\left(x_{\left(i,2\right)}\right)\right]}^{2}}$$

which corresponds to the root mean square of the differences between transformed and original control points [44,45]. The same method is applied to calculate the registration error along the y-axis (${FRE}_{y}$), and the total FRE is the quadratic sum of ${FRE}_{x}$ and ${FRE}_{y}$. To determine the optimal scanning step size—defined as the one yielding the lowest FRE—five calibration acquisitions were performed for each step size. By comparing the resulting FRE values, it was found that step sizes of 600 nm and 900 nm had the lowest median FREs (see Supplementary Figure S5). However, the 900 nm step size exhibited greater variability (i.e., higher standard deviation), so 600 nm was selected as the optimal optical scanning step size. The FRE associated with this step size is 3.5 nm.

For 3D acquisitions, chromatic aberration correction was performed in two steps. First, axial chromatic aberration was corrected by estimating the axial shift between the two-color channels from calibration beads, yielding a linear focal shift of 9.8 nm that was applied as a global correction (Supplementary Figure S6). Second, a full 3D chromatic aberration correction was implemented to account for lateral (x–y) shifts as a function of the axial position, following an approach analogous to the 2D case. A 3D scan of fluorescent calibration beads was acquired, and a 3D geometric transformation between color channels was computed using a custom MATLAB algorithm based on the fitgeotform3D function. An axial step size of 600 nm, which minimized the Fiducial Registration Error (Supplementary Figure S5), was used. The central region of the field of view was scanned using a 24 × 27 × 3 grid, yielding 1944 calibration points while limiting bead photobleaching. The resulting 3D calibration corrects chromatic aberration over an axial range of ±600 nm, matching the astigmatism calibration range (Supplementary Figure S7), and achieves a mean registration error of 11 nm.

### 2.4. Crosstalk Correction

Optical crosstalk in multicolor imaging happens when the signal from one emitter is detected in the channel of the other emitter due to overlapping of excitation and emission spectra that causes inaccurate spectral separation. This contamination can lead to unreliable results, such as false positives or negatives. In single-molecule co-localization, a careful selection of spectrally separated dyes and the use of narrow bandpass filters can be insufficient to effectively reduce the impact of crosstalk. For this reason, a custom Matlab algorithm was developed to correct crosstalk at the pixel level for each frame by comparing the gray values of corresponding pixels in the two channels. With the adopted optical configuration, a crosstalk signal of PAGFP was detected in the PAmCherry1 channel (as shown in Supplementary Figure S8). To account for this effect, before each acquisition set, we acquire several frames with only PAGFP activated to record its signal in both channels. By comparing the signals recorded in both channels, the algorithm estimates the percentage of crosstalk and corrects for it. First, the algorithm estimates a non-rigid transformation function to align the two channels based on pixel coordinates of grid points. Next, it calculates the *crosstalk factor* (CF), pixel by pixel, as:

(2) $$CF=\;\frac{I_{Ch2\;crosstalk}}{I_{Ch1}}$$

where $I_{Ch1}$ is the intensity of the emitted signal recorded in channel 1, while $I_{Ch2\;crosstalk}$ is the intensity of the crosstalk signal recorded in the channel 2. CF is an estimate of the crosstalk signal for every pixel of each frame. The corrected intensity values are then calculated as:

(3) $$I_{Ch2\;corrected}=I_{Ch2\;measured\;}-\;CF\;\cdot I_{Ch1}$$

where $I_{Ch2\;measured\;}$ is the original recorded intensity. This correction is applied pixel by pixel for the channel affected by crosstalk for recorder intensities above the average background.

### 2.5. E. coli Expansion Protocol

The complete protocol, including all reagents and their concentrations for expanding *E. coli* bacteria, is reported in Supplementary Protocols. This protocol was optimized from Fan et al. [46] and Middelhauve et al. [47]. First, bacteria are grown in M9 culture medium with 0.4% glucose, 25 µg/mL histidine, 50 µg/mL IPTG, and 50 µg/mL kanamycin for 31 h at 37 °C with shaking at 215 rpm. Then, cells are collected by centrifugation, washed in PBS, and fixed in 4% PFA for 10 min. After washing with PBS, the optical density at 600 nm (OD600) is measured to ensure it falls within 1.8–2.1. Then, cells are permeabilized with PBSTx and further treated with 50% methanol in PBSTx. Due to increased fragility after permeabilization, all subsequent centrifugations are performed at 2000 *g*. Cell walls are then digested overnight at 37 °C with 320 units/mL mutanolysin, washed in PBS, and then incubated with 2 mM MA-NHS in PBS for chemical anchoring. After additional PBS washes, the sample are mixed with a gelation solution and then incubated in a humid chamber for 2 h at 37 °C. The gel is then digested in a solution containing 8 units/mL Proteinase K for 3 h, causing an initial ~1.5-fold expansion. The gel is subsequently transferred to a Petri dish and further expanded in MilliQ water, which is either replaced every 15 min for 45 min or left overnight at 4 °C. This step leads to a final isotropic expansion factor of approximately 4×. For imaging, the expanded gel is cut into small pieces, mounted on a glass coverslip coated with 0.1% poly-L-lysine, and kept hydrated with water to prevent drying.

The optimization of the expansion protocol involved several key adjustments. First, the concentration of mutanolysin was carefully calibrated to ensure complete digestion of the bacterial cell wall, which is essential to achieve a uniform expansion. Second, the concentration of MA-NHS had to be adjusted to effectively anchor proteins to the gel, since a low concentration resulted in poor protein retention and imaging quality, while a high concentration made the gel too rigid, causing non-uniform expansion. Third, the duration of the digestion step was adjusted according to the increased MA-NHS concentration, thus ensuring complete sample digestion and isotropic expansion while avoiding bacterial rupture and unwanted background signals caused by dispersed chromophores. The success of the expansion process depends on the biological sample (e.g., tissue, eukaryotic cells, or prokaryotic cells) and the fluorescent probes used for protein labeling, as different fluorescent proteins respond differently to treatments. By finely tuning these parameters, dual-color imaging of PAGFP and PAmCherry1 in *E. coli* bacteria was achieved.

### 2.6. Multicolor Ex-PALM Imaging

To perform Ex-PALM imaging, a fixed sample of expanded bacteria in a polyelectrolyte gel was prepared as described above. As with standard PALM imaging, a calibration sample with fluorescent beads was imaged prior each acquisition for chromatic aberration correction. Ex-PALM multicolor acquisition was performed at 80 ms exposure time and 400 EM Gain. To improve the contrast, which is fundamental to detect single molecules in thick samples [40], the sample was excited with an inclined beam that illuminates a portion of the sample with an angle of 69° and a thickness of 3.8 μm (as shown in Supplementary Figure S2) in the custom setup described in the Section 2.2. By exciting a thin slice of the sample, the background signal from the out-of-focus portion of the sample is decreased, thus greatly improving signal-to-background ratio and, consequently, improving single-molecule detection performance [40]. All lasers were turned on simultaneously at the same power settings used for non-expanded bacteria, with a duration of about 80 s for each acquisition.

### 2.7. Localization of Single Molecules in Multicolor Imaging

The analysis of multicolor PALM/Ex-PALM images to achieve super-resolution maps of labeled proteins consists of four main steps: (i) crosstalk correction, (ii) single-molecule localization, (iii) chromatic aberration correction, and (iv) merging of reappearing molecules.

**Figure 2:**
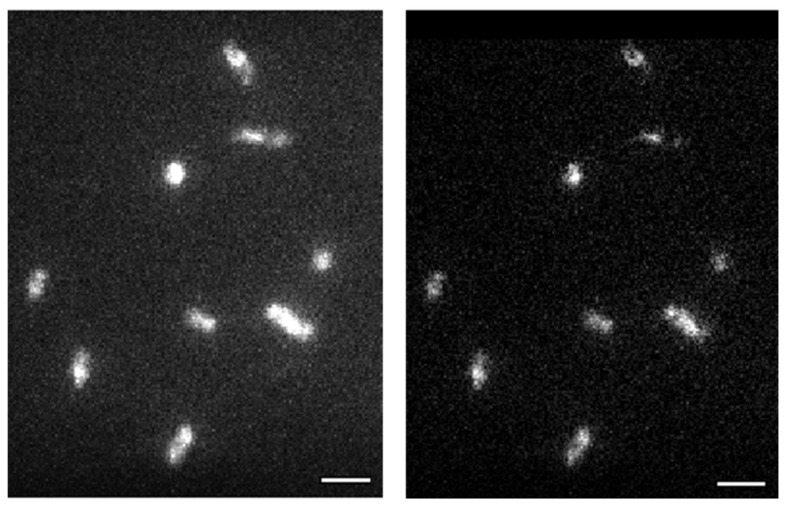
Example of a frame in the channel of PAmCherry1 before (**left**) and after (**right**) crosstalk correction. Scalebar: 2 µm.

First crosstalk correction is performed using the custom algorithm described in the Section 2.4 and in Figure 2.

Then, single-molecule localization is achieved for both PAGFP and PAmCherry1, by means of the ThunderSTORM 1.3 [48] ImageJ 1.54p plugin (see Supplementary Figure S9 for information on the parameters). Localization precision was estimated using repeated localizations, defined as molecules that reappeared within ten frames from their initial detection, at distances smaller than the mean Thompson uncertainty. Following Huang et al.’s approach [41], for each molecule, localizations were aligned to their center of mass to generate a 2D or 3D representation of the localization distribution, depending on the imaging technique (2D PALM, 2D Ex-PALM, or 3D Ex-PALM). Histograms of the localization distributions along each axis were fitted with Gaussian functions, and the localization precision for each axis was obtained from the standard deviation of the corresponding fitting function. The 2D and 3D localization distributions, along with the localization precision for each axis, are shown in Supplementary Materials Figures S10–S12, while the mean values are reported in Table 1.

**Figure 3:**
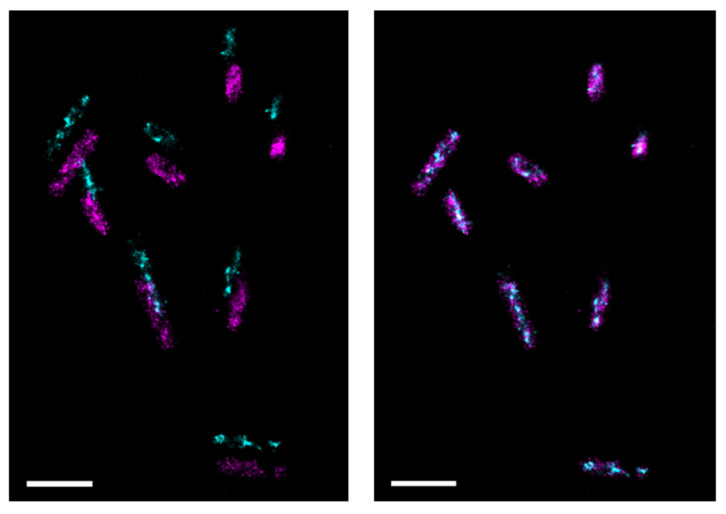
Example of an acquisition before (**left**) and after (**right**) chromatic aberration correction. Cyan points are localizations of PAGFP while magenta points are localizations of PAmCherry1. Scalebar: 2 µm.

Chromatic aberration correction is then applied to single-molecule localization, as described previously. Figure 3 shows an example of before and after correction. As clearly visible in the image, chromatic aberrations cause a positioning error of hundreds of nanometers between the two channels. Since intermolecular distances are on the order of a few tens of nanometers, it would be impossible to obtain reliable measurements without fine correction. The algorithm achieves a registration error of few nm—specifically, 3.5 nm for PALM acquisitions, 1.0 nm for 2D Ex-PALM acquisitions, and 11 nm for 3D Ex-PALM acquisitions—enabling reliable reconstruction of the spatial organization proteins in bacteria.

Finally, the merging of reappearing molecules is applied to prevent overcounting of the same photoactivated molecule. In single-molecule experiments, it can happen that the same molecule reappears for several frames before permanently photobleaching. For each localized molecule, a nearest-neighbor search identifies as duplicates all the localizations closer than the mean value of Thompson’s uncertainty [49] (about 20 nm for PALM and about 10–13 nm for Ex-PALM) and separated by less than 10 frames from the initial appearance. Duplicates are merged into a single molecule using intensity-weighted average coordinates, and the localization uncertainty is determined as the quadratic sum of standard deviations of duplicate positions along the three axes [50].

### 2.8. Co-Localization Analysis

Once the maps of localizations are obtained, co-localization analysis can be performed. In conventional fluorescence microscopy, co-localization is typically quantified using parameters such as Pearson’s correlation coefficient [51] or Manders’ overlap coefficients [52], which measure the degree of overlap between pixel intensities across an image. However, in single-molecule localization microscopy (SMLM), co-localization is defined differently, as single emitters rarely occupy the exact same position. Instead, it is quantified in terms of intermolecular distance or spatial association.

Through the years, several methods have been developed for SMLM co-localization analysis, including spatial point pattern analysis [53,54,55] and cluster-based approaches [56,57,58]. In this study, the local density-based co-localization index introduced by Willems et al. [59] was employed. This index evaluates co-localization by measuring the local density of molecules in one channel around localizations in the other channel (as shown in Supplementary Figure S13). For each $i^{th}$ localization in channel A, the co-localization index (${CI}_{i}^{A}$) is defined as:

(4) $${CI}_{i}^{A}=\;\frac{N_{Ai}^{B}(d_{B})}{{\bar{LD}}_{B}}$$

which is the ratio of the number of molecules in channel B within a specified distance ($N_{Ai}^{B}$) to the mean local density of molecules in channel B (${\bar{LD}}_{B}$). Similarly, the co-localization index for channel B is calculated based on molecules in channel A (${CI}_{i}^{B}$). This parameter provides a measure of the similarity in spatial distributions between channels. Co-localizing pairs of molecules are identified by calculating the co-localization index for all localizations in both channels. Couples having a co-localization index greater than zero are considered as co-localizing pairs. Then, with a nearest-neighbor search, the distance between co-localizing and not co-localizing pairs is calculated, and finally, a mean distance between all the pairs of molecules in the sample is calculated as:

(5) $$d=\;\frac{N_{c}\cdot d_{c}+N_{nc}\cdot d_{nc}}{N_{c}+N_{nc}}$$

where $N_{c}$ and $N_{nc}$ are, respectively, the number of co-localizing and not co-localizing pairs, while $d_{c}$ and $d_{nc}$ are, respectively, the mean distance between co-localizing and not co-localizing pairs. By calculating the average distance including both co-localizing and non-co-localizing couples, d should allow one to discriminate samples with randomly distributed proteins from samples with co-localizing proteins, as is the case of the two *E. coli* strains described above.

**Figure 4:**
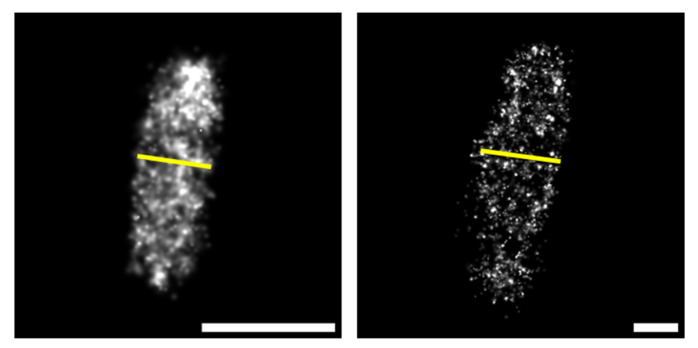
Estimation of the expansion factor. Measurement of the average width of an unexpanded (**left**) and expanded (**right**) bacterium. The yellow line represents the width of the bacterium, which was used to estimate the expansion factor, as the width can be measured precisely regardless the cellular orientation in the three dimensions. Scalebar: 1 µm.

For Ex-PALM acquisitions, the expansion factor was estimated prior to measuring the intermolecular distances. This parameter was calculated by estimating the average width of bacteria before and after the expansion (as shown in Figure 4), as the width can be measured precisely regardless of the cellular orientation in the three dimensions [60,61]. We obtained an average expansion factor of 3.97 ± 0.57 for PHPF and of 3.86 ± 0.57 for PHPA ($N_{unexp}=10,\;N_{expPHPF}=10,$ $and\;N_{expPHPA}=10$).

## 3. Results

### 3.1. Multicolor PALM Imaging

To validate the experimental procedure described before, it was first applied to both simulated and real 2D PALM images. Simulations were performed using Single-Molecule Imaging Simulators (SMIS) [62], a Matlab software specifically developed for simulating single-molecule localization microscopy experiments. A multicolor PALM experiment of two photoactivable fluorescent proteins with spectra and photoswitching properties corresponding to PAGFP and PAmCherry1 inside *E. coli* cells was simulated. A “positive control” sample (corresponding to the real case of PHPF), where the photoswitching proteins co-localize with an average distance of 10 ± 5 nm, was simulated, as well as a “negative control” (corresponding to the real case of PHPA), where the photoswitching proteins were randomly distributed within the bacterial cell volume. Importantly, the molecular density of fluorophores inside the bacteria was set to match a realistic value of about 6800 molecules/µm^3^—calculated considering the mean size of bacteria and the average number of localizations per bacterium we usually measure—as the probability of having false positive co-localizations increases with increasing density (as shown in Supplementary Figure S14).

**Figure 5:**
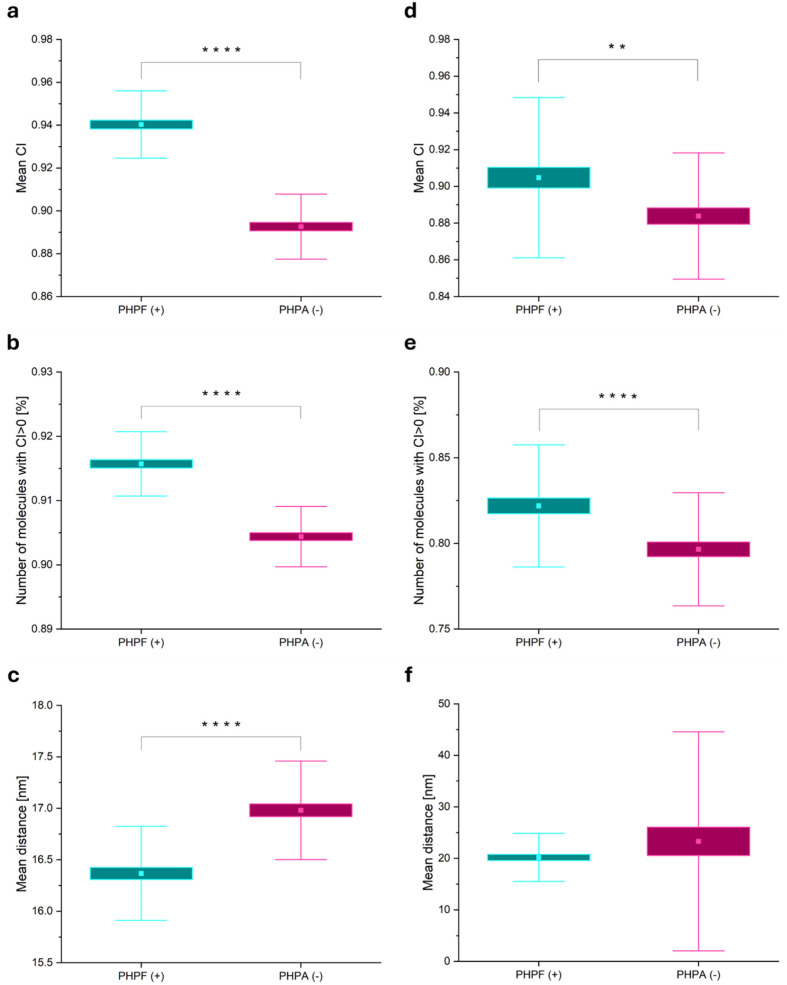
Results of co-localization analysis of multicolor PALM imaging. (**a**–**c**) Simulations: Box plots showing (**a**) mean co-localization indices (CIs), (**b**) percentage of co-localizing molecules, and (**c**) mean distance between all pairs of molecules. Data were derived from 60 simulations of positive (PHPF, N = 60, cyan box plot) and negative (PHPA, N = 60, magenta box plot) control samples. (**d**–**f**) Analysis of real acquisitions: Box plots showing (**d**) mean CIs, (**e**) percentage of co-localizing molecules, and (**f**) mean distance between all pairs of molecules. Data were derived from 60 acquisitions of positive (PHPF, N = 60, cyan box plot) and negative (PHPA, N = 60, magenta box plot) control samples. In each box plot, the square represents the mean value, the box is ±1 SE, and the whiskers are ±1 SD. ****: *p* < 0.0001 two-sample *t*-test; **: *p* < 0.01 two-sample *t*-test. Summarized data can be found in Supplementary Table S1.

Figure 5 summarizes the results of the co-localization analysis on the two simulated experiments. From the analysis, the following features were extracted for the quantification of the degree of co-localization and the distances between molecules pairs: the mean co-localization index (CI), the percentage of co-localizing molecules (i.e., molecules with CI>0), and the mean distance (d). As discussed in the previous sections, the CI can be defined for each acquisition channel. For clarity, the CI of the PAGFP channel is reported. Figure 5a shows the mean CI of PAGFP of positive (PHPF) and negative (PHPA) control samples, respectively. A two-sample *t*-test confirmed that for the “negative control”, CIs were significantly lower than those for “positive control” datasets (*p* < 0.0001). Figure 5b shows the percentages of co-localizing molecules. Although these percentages are high (>90%) in both samples due to the high molecular density of fluorophores, which leads to many false positive co-localizations among randomly distributed proteins, a significant difference (*p* < 0.0001) between the positive and negative control samples was found. This result validates the CI as a robust indicator to discern different degrees of co-localization even in very dense samples. Finally, the mean distances between all the pairs in the sample (from Equation 5) are shown in Figure 5c. As expected, pairs of molecules in randomly distributed proteins have a greater mean distance of 16.37 ± 0.06 nm, with respect to that of co-localizing proteins, which was 16.98 ± 0.06 nm. Also in this case, although the high molecular density leads to many false positive co-localizations in the negative control sample, a significant difference (*p* < 0.0001) in the mean distances between all pairs within the samples was found.

Then, real data acquisitions taken from the “positive control” strain (PHPF) and “negative control” strain (PHPA) were analyzed. Figure 5d reports the mean CIs of both positive and negative control samples. Consistent with simulated data, negative control CIs were significantly lower than positive ones (*p* < 0.01), even if the distributions showed a greater variability compared to simulations. It has to be taken into account that with respect to simulations, in real bacteria, *hisH* and *hisF*/*aac(3)IV* genes are located on the same plasmid molecules and organized in a bicistronic operon. Hence, the two coding sequences in the principle may permit an initial co-localization of non-interacting proteins (HisH and apramycin acetyl transferase). Figure 5e shows the percentage of co-localizing molecules, which is significantly higher in the positive control (*p* < 0.0001). Figure 5f reports the mean distances between pairs (from Equation 5), corresponding to 20.2 ± 0.6 nm for PHPF and 23 ± 3 nm for PHPA. These values are consistent with expected intermolecular distances.

However, differently from simulation results, in this case, there was no significant difference between samples due to high variance in the data.

Overall, this first analysis proved that the proposed co-localization imaging procedure is effective in calculating intermolecular distances and discerning co-localizing pairs against randomly distributed ones. However, achieving higher resolution is essential for gaining deeper insights into bacterial subcellular organization, since in 2D PALM images, distances below 12 nm could not be measured, and the axial coordinate was not localized. A combination of 3D PALM and ExM was applied to improve these limitations.

### 3.2. Multicolor Ex-PALM Imaging

**Figure 6:**
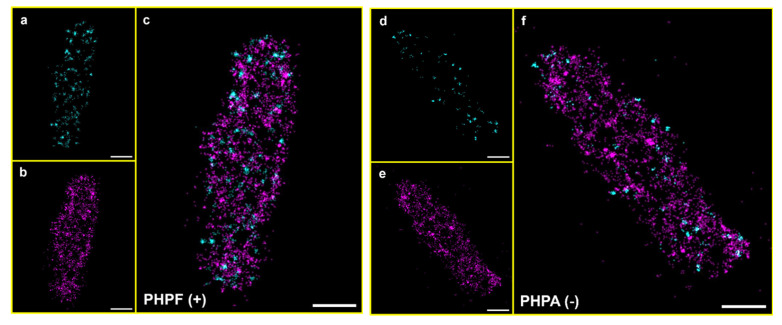
Expanded *E. coli* bacterium reconstructed from a 2D Ex-PALM acquisition. (**a**–**c**) PHPF sample: PAGFP localizations (cyan), PAmCherry1 localizations (magenta), and merged channels. (**d**–**f**) PHPA sample: PAGFP localizations (cyan), PAmCherry1 localizations (magenta), and merged channels. Scalebar: 1 µm.

**Figure 7:**
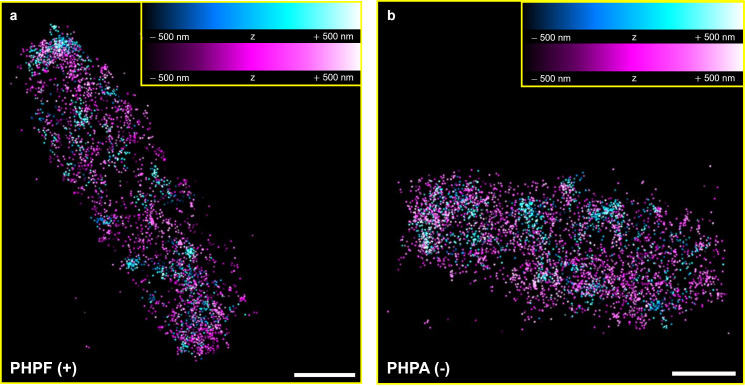
Merged channels of an expanded *E. coli* bacterium reconstructed from a 3D Ex-PALM acquisition. (**a**) PHPF sample and (**b**) PHPA sample. PAGFP localizations are shown in a hot cyan color scale and PAmCherry1 localizations in a hot magenta color scale, with colors encoding axial position (z). Axial range: ±500 nm. Scalebar: 1 µm.

As discussed in Section 2.8, the ExM treatment produced an isotropic expansion of bacteria by approximately 4 times in all the three dimensions, resulting in a volume increase of about 64 times compared to unexpanded bacteria. A direct consequence is a decrease of the molecular density by the same factor, making three-dimensional single-molecule localization through astigmatism possible. Figure 6 and Figure 7 and Supplementary Figure S17 show examples of reconstructed images of expanded bacteria acquired using, respectively, 2D and 3D Ex-PALM imaging.

**Figure 8:**
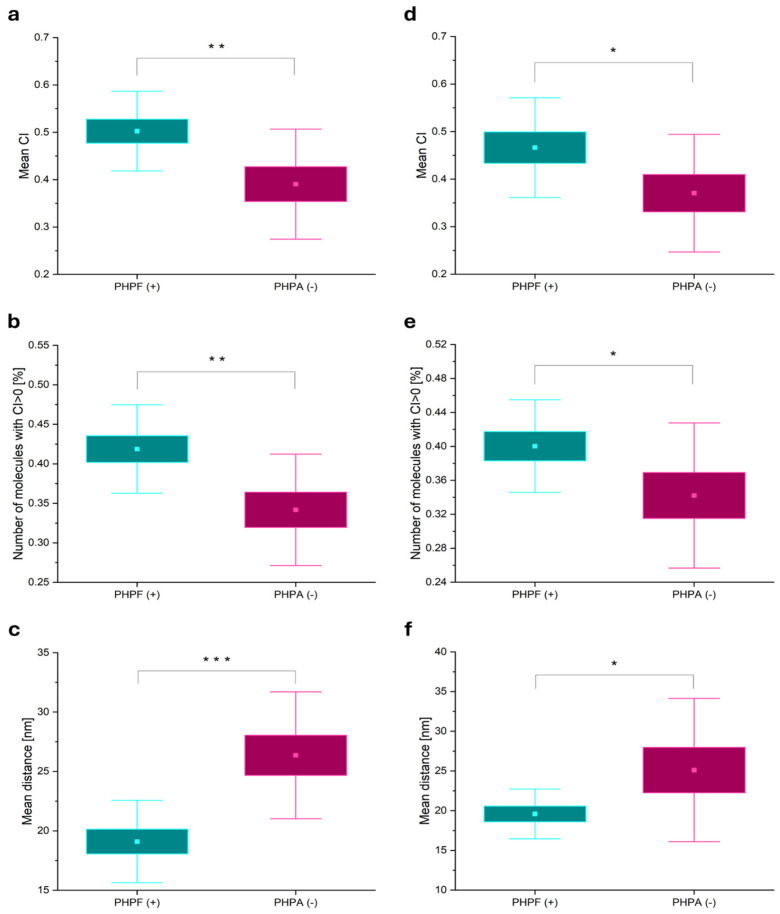
Results of co-localization analysis on multicolor Ex-PALM acquisitions. (**a**–**c**) 2D Ex-PALM: Box plots showing (**a**) mean co-localization indices (CIs), (**b**) percentage of co-localizing molecules, and (**c**) mean distance between all pairs of molecules. Data were derived from 10 acquisitions of positive (PHPF, N = 10, cyan box plot) and negative (PHPA, N = 10, magenta box plot) control samples. (**d**–**f**) 3D Ex-PALM: Box plots showing (**d**) mean CIs, (**e**) percentage of co-localizing molecules, and (**f**) mean distance between all pairs of molecules. Data were derived from 10 acquisitions of positive (PHPF, N = 10, cyan box plot) and negative (PHPA, N = 10, magenta box plot) control samples. In each box plot, the square represents the mean value, the box is ±1 SE, and the whiskers are ±1 SD. ***: *p* < 0.001 two-sample *t*-test; **: *p* < 0.01 two-sample *t*-test; *: *p* < 0.05 two-sample *t*-test. Data are summarized in Supplementary Table S2.

In Figure 8, the results of 2D and 3D Ex-PALM image analysis are reported. As for PALM images, the following parameters were retrieved from each acquisition: (i) the mean CI, (ii) the percentage of co-localizing molecules, and (iii) the distance between all pairs of molecules. For 2D EX-PALM, Figure 8a shows the mean CIs of PAGFP. Consistent with previous results, the CIs of the negative control were significantly lower than those of the positive control (*p* < 0.01). However, compared to PALM acquisitions, the average CIs were lower. This is probably due to fluorophore loss during expansion, with PAGFP being more affected than PAmCherry1 (as shown in Supplementary Figures S15 and S16). Figure 8b shows the percentage of co-localizing molecules, which was also lower with respect to PALM acquisitions but remained significantly higher in positive controls (*p* < 0.01). This decrease was also attributed to protein loss during expansion. Figure 8c reports mean distances between all pairs of molecules in the sample (from Equation (5)), corrected by the expansion factors (3.97 for PHPF; 3.86 for PHPA), yielding values of 19.1 ± 1.0 nm for PHPF and 26.4 ± 1.7 nm for PHPA. Unlike 2D PALM acquisitions, in this case, significant differences were found in the mean distances between the samples (*p* < 0.001), thanks to the increased resolution achieved by combining ExM biological treatment with the PALM imaging process. As explained in Section 2.8, the mean distance is calculated by considering the distances between all pairs in the sample, including both co-localizing and non-co-localizing pairs. Thanks to the enhanced resolution of 2D Ex-PALM, distances were measured more accurately in both samples. It was found that co-localizing pairs have a distance of 6.8 ± 0.2 nm for PHPF and 8.6 ± 0.5 nm for PHPA. These values were not only significantly lower than those obtained with 2D PALM (12.52 ± 0.08 nm for PHPF and 12.6 ± 0.1 nm for PHPA) but also showed a clear difference between the two samples. The significant difference in co-localizing distances between the two samples leads to a significant difference in the mean distance between all pairs. This result demonstrates that, on average, interacting proteins tend to be closer to each other than proteins that are merely nearby in space.

For 3D Ex-PALM, Figure 8d–f report the mean CIs of PAGFP, the percentage of co-localizing molecules, and the 3D mean distances of all the pairs, respectively. For both the CI and the percentage of co-localizing molecules, significantly higher values were observed in positive controls than in negative controls (*p* < 0.05), while for the mean distance, significantly lower values were observed for PHPF with respect to PHPA, with consistency between 3D Ex-PALM and 2D Ex-PALM values. The average distances (in the 3D) were 19.6 ± 1.0 nm for PHPF and 25 ± 3 nm for PHPA.

**Figure 9:**
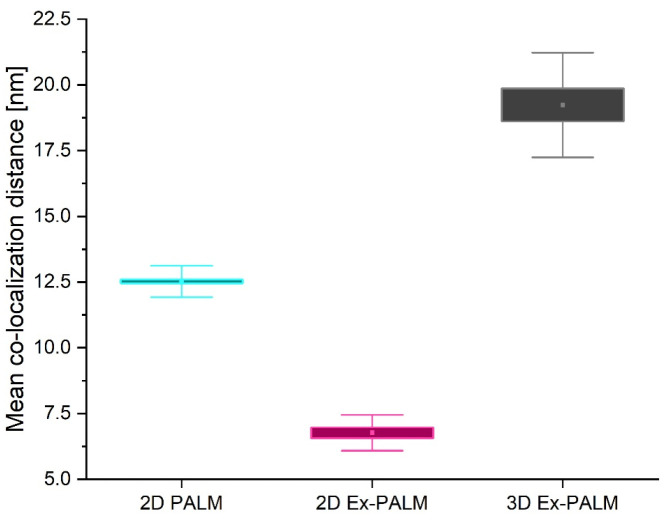
Comparison of the mean co-localization distance of interacting proteins (PHPF) obtained with different techniques: 2D PALM (N = 60, cyan box plot), 2D Ex-PALM (N = 10, magenta box plot), and 3D Ex-PALM (N = 10, black box plot). In each box plot, the square represents the mean value, the box is ±1 SE, and the whiskers are ±1 SD.

Finally, the mean co-localization distances that can be measured between interacting pairs using the three different techniques (2D PALM, 2D Ex-PALM, and 3D Ex-PALM) are reported in Figure 9. We found mean co-localization distances of 12.52 ± 0.08 nm for 2D PALM, 6.8 ± 0.2 nm for 2D Ex-PALM, and 19.2 ± 0.6 nm for 3D Ex-PALM. These results confirm the improved resolution achieved by combining the two techniques in the 2D configuration, enabling measuring distances down to 6 nm. On the other hand, they also demonstrate that Ex-PALM enabled 3D single-molecule imaging in a crowded environment with a minimum measured distance that is compatible with expected molecular distances base on proteins steric hindrance.

## 4. Discussion

In this work, a technique for super-resolution multicolor imaging of *E. coli* cells with reliable results was developed. This was achieved through three key improvements: (i) the minimization of main multicolor imaging aberrations, (ii) the combination of ExM with PALM, and (iii) the greatly improved signal-to-background ratio achieved with ultra-confined illumination achieved with a custom single-objective light sheet. First, the achievable localization precision in multicolor PALM was optimized by minimizing aberrations. These corrections enabled us to study the spatial distribution of proteins in *E. coli* cells. The consistency between simulation and acquisition results demonstrated that the proposed method allows for super-resolution co-localization measurements in small, crowded volumes with nanometer precision. Using this approach, positive and negative control samples were successfully distinguished based on the co-localization index and the percentage of co-localizing molecules. However, due to localization and registration errors that limited the resolution of our setup to about 12 nm, no significant difference in the mean distance between molecular pairs could be observed. Moreover, the high molecular density of fluorophores in the small bacterial volume prevented three-dimensional imaging, as the PSFs of the emitters overlapped and became undistinguishable. By combining Expansion treatment with PALM and confined inclined illumination, three-dimensional single-molecule localization in *E. coli* was achieved, with a localization precision of 3 nm in the three dimensions. Under these conditions, a significant difference between the mean distance of interacting and non-interacting proteins was observed, and a minimum distance of few nanometers was measured (a flow chart of the whole procedure can be found in Supplementary Figure S20).

A further step towards the comprehension of molecular compartmentalization in *E. coli* could be carried out by applying our method to native HisF and HisH proteins replaced by their fluorescent counterparts, PAmCherry1-HisH and PAGFP-HisF, respectively, to directly observe their physiological expression levels under their native promoter. The same approach could be extended to other pairs of proteins of interest and further applied to the study of DNA folding in bacteria. With appropriate labeling of selected sequences, super-resolution maps of DNA elements (e.g., chromosomes, chromosomal regions, and plasmids) could be obtained.

By integrating molecular specificity with super-resolution and physical expansion, this technique represents an effective tool for studying molecular organization in *E. coli* and, with appropriate adjustments, could be applied to high-density samples in general, with broad applications in microbiology, synthetic biology, and cellular biophysics.

## 5. Conclusions

A strategy for multicolor nanoscale imaging in *E. coli*, integrating PALM, Expansion Microscopy, and ultra-confined illumination using a custom single-objective light sheet, is presented. By minimizing multicolor aberrations and improving the signal-to-background ratio, reliable co-localization analysis within the crowded intracellular environment was enabled.

While conventional multicolor PALM allowed for discriminating between interacting and non-interacting protein pairs through co-localization metrics, the achievable localization precision of approximately 12 nm limited direct distance measurements. These limitations were overcome by integrating Expansion Microscopy with PALM, enabling three-dimensional single-molecule localization in *E. coli* with a precision of approximately 3 nm and revealing significant differences in intermolecular distances at the nanometer scale.

This approach establishes a general framework for probing molecular organization in bacteria and provides a route toward quantitative nanoscale studies of protein interactions and DNA organization in highly crowded cellular systems.

## Figures and Tables

**Table 1:** Mean localization precision values of PAGFP and PAmCherry1 obtained using different imaging techniques.

	Localization Precision		
	2D PALM	2D Ex-PALM	3D Ex-PALM
**PAGFP**	$∆\bar{xy}=$ 6.7 nm	$∆\bar{xy}=$ 1.7 nm	$∆\bar{xy}=$ 3.2 nm $∆\bar{z}=$ 3.3 nm
**PAmCherry1**	$∆\bar{xy}=$ 5.8 nm	$∆\bar{xy}=$ 1.6 nm	$∆\bar{xy}=$ 2.6 nm $∆\bar{z}=$ 2.8 nm

## Data Availability

Example raw data are shared as Supporting Information. More data will be available upon request. Custom scripts used in this study are available at https://github.com/caldini-c/Ex-PALM, accessed on 14 January 2026.

## Supplementary Materials

The following supporting information can be downloaded at: https://www.mdpi.com/article/10.3390/microorganisms14040772/s1, Figure S1: Maps of plasmids pSEVA224-PHPF (top) and pSEVA224-PHPA (bottom) used to transform E. coli FB1 cells. Figure S2: Scheme of the experimental setup. Figure S3: Step by step assembly protocol of the agaro se pad with embedded bacteria used for 2D PALM acquisitions. Figure S4: Main steps of the algorithm used to correct for chromatic aberrations. Figure S5: Comparison of FRE between different scanning step sizes. In each box plot, the square represents the mean value, the box is ±1 SE and the whiskers are ±1 SD. Figure S6: Estimate of the focal shift between the two channels due to axial chromatic aberration. Figure S7: Example of the calibration grid used to estimate the transformation function and correct chromatic aberration in 3D. Figure S8: Transmittance as a function of wavelength of fluorescent proteins (PAGFP and PAmCherry1) and detection optical elements (dichroic mirrors and fluorescent filters) of our setup. Figure S9: ThunderSTORM user interface showing the parameters used to find the sub-pixel positions of single molecules of PAGFP and PAmCherry1 in bacteria in simulations, 2D PALM and 2D Ex-PALM (left) and 3D Ex-PALM (right). Figure S10: Localization precision of PAGFP (cyan, N = 25,122) and PAmCherry1 (magenta, N = 39,505), estimated from repeated localizations of the same molecules obtained from ten 2D PALM acquisitions. Figure S11: Localization precision of PAGFP (cyan, N = 2767) and PAmCherry1 (magenta, N = 6876), estimated from repeated localizations of the same molecules obtained from ten 2D Ex-PALM acquisitions. Figure S12: Localization precision of PAGFP (cyan, N = 778) and PAmCherry1 (magenta, N = 2173) , estimated from repeated localizations of the same molecules obtained from ten 3D Ex-PALM acquisitions. Figure S13: Concept of local density-based co-localization index. Figure S14: Relation between molecular density of fluorophores and co-localization analysis. Figure S15: Protein fluorescence signal retention after expansion. Figure S16: Protein retention after expansion. Figure S17: Orthogonal projections of the 3D Ex-PALM acquisitions shown in Figure 7 of the manuscript. Table S1: Results of the co-localization analysis of multicolour PALM simulations and acquisitions. For each parameter is reported the mean value and its standard error. Table S2: Results of the co-localization analysis of multicolour 2D and 3D Ex-PALM imaging. For each parameter is reported the mean value and its standard error. Figure S18: Two-channel chamber composed of a rectangular coverslip attached on a microscope slide through thin strips of double-sided tape, which also delimit the channels. Table S3: Scanning parameters for different scanning step sizes covering a field of view of around 38 × 16 μm^2^. Figure S19: Assembling of the gelation chamber. Figure S20: Flowchart summarizing the experimental workflow from sample preparation to co-localization analysis.
