# Supplementary material for: 3D Expansion–PALM (PhotoActivated Localization Microscopy) Dissects Protein–Protein Interactions Down to the Molecular Scale in Bacteria
Source: Microorganisms. 2026 Mar 28;14(4):772. doi: 10.3390/microorganisms14040772 (PMC13119062; doi:10.3390/microorganisms14040772)
Supplement: Supplementary file 1 [file microorganisms-14-00772-s001.zip › microorganisms-4208856-supplementary.pdf]

Supplementary Information

Supplementary Figures

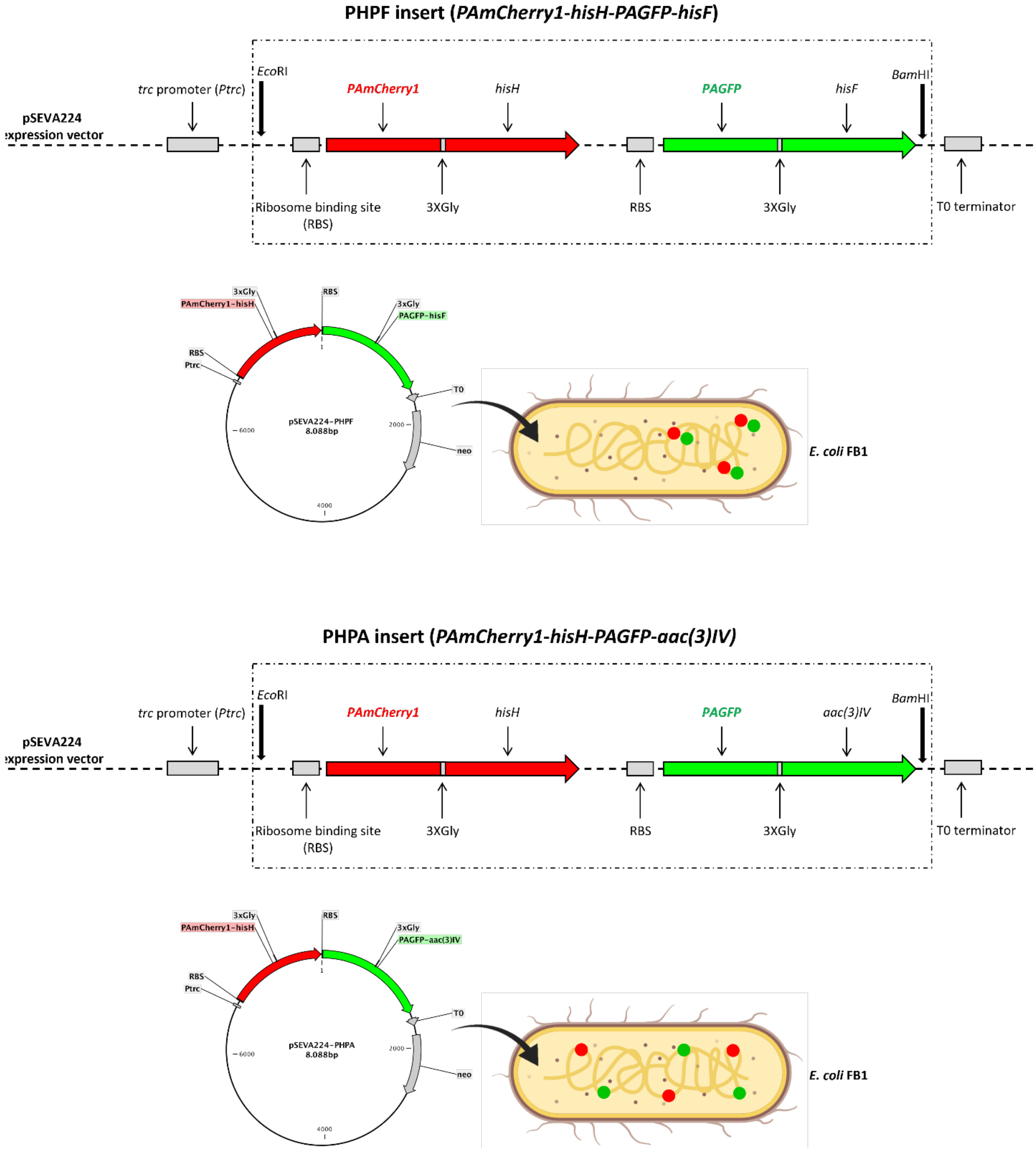

Figure S1: *Maps of plasmids pSEVA224-PHPF (top) and pSEVA224-PHPA (bottom) used to transform E. coli FB1 cells. The two inserts (PHPF and PHPA) synthesized by the external company Bio-Fab Research s.r.l. (Rome, Italy) are outlined with dashed lines.*

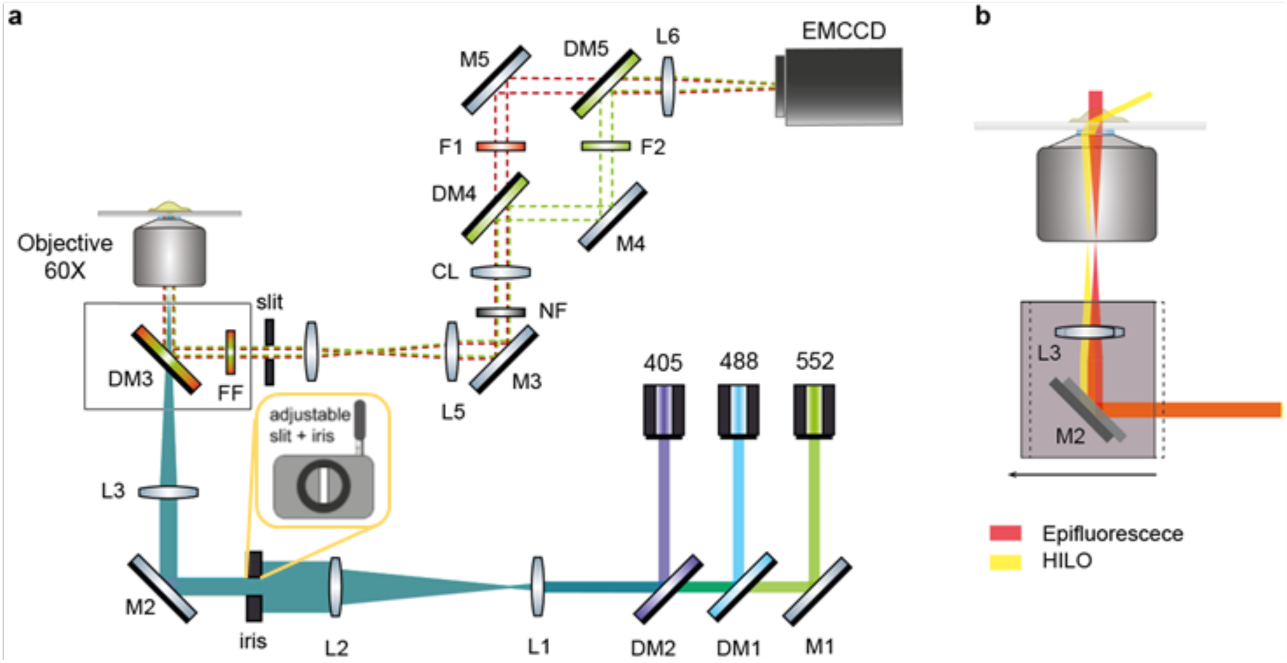

Figure S2: *Scheme of the experimental setup. Panel (a) depicts the complete setup, whereas (b) outlines the available excitation methods, which include epifluorescence and HILO. The experimental setup consists of a Nikon ECLIPSE TE300 inverted fluorescence microscope with three diode lasers with wavelengths of 552 (Coherent, OBIS LS 552 nm 100 mW), 488 (Coherent, OBIS LS 488 nm 100 mW) and 405 (TOPTICA, iBeam smart PT 405 60 mW) for excitation and an EMCCD camera (Andor iXon X3) for detection. The excitation beam from the laser is either reflected by a mirror (M1 for 552 nm) or a dichroic mirror (DM1/DM2 for 488/405 nm) and directed towards the telescope L1 (f = 50 mm) – L2 (f = 500 mm), which magnify it by a factor of 10. After the telescope, a circular iris is placed in a plane conjugated with the image plane to adjust the size of the field of view, ensuring uniform illumination across the entire field. Then, the beam is reflected by mirror M2 and focused through lens L3 (f=500 mm) into the back focal plane of a Nikon 60x oil-immersion TIRF objective with 1.49 NA. Both the mirror M2 and the lens L3 are mounted on linear motorized translators (Physik Instrumente, M-014.D01 and M-126.CG), that enables adjusting the angle of incidence of the excitation light and thus different illumination configurations (epifluorescence or HILO). In HILO configuration the thickness of the inclined beam can be regulated down to 3 microns at the sample plane through a linear slit in a conjugated plane with the image[1,2]. The emitted fluorescence is collected by the objective mounted in an inverted configuration, separated from excitation light by the dichroic mirror DM3 (Chroma, ZT488/561rpc-uf2), filtered with the multi band-pass filter FF (Semrock, FF01-515/588/700-25), magnified by a factor of 3 thanks to the telescope L5 (f = 50 mm) – L6 (f = 150 mm) and projected onto the EMCCD camera, yielding a 42 × 42 μm^2 field of view corresponding to a pixel size of 82 nm. A notch filter, F2 (Semrock, NF03-405/488/561/635E-25), is placed in the detection path to attenuate laser wavelengths by approximately seven orders of magnitude, ensuring that only fluorescence emission reaches the detector. This is crucial for single-molecule fluorescence measurements, where minimizing background signals is essential. Simultaneous two-colour imaging is achieved by using a rectangular slit to split the field of view and select only one half, two identical dichroic mirrors (Semrock, LM01-552-25.0x35.6) and short band filters (Semrock, FF01-511/20-25 and FF01-595/31-25) to separate the emissions of the two fluorophores and direct them to the detector. 3D imaging is performed by inserting a cylindrical lens in the detection path that introduces astigmatism, encoding z-positions without significantly degrading lateral resolution[3]. Both the sample and objective are mounted on piezoelectric stages for precise xyz positioning (Physik Instrumente, P-721.C PIFOC and P-527.2CL). The entire system is controlled via a custom LabVIEW program developed by our group[4], with active stabilization to minimize mechanical and thermal drifts, enabling long-term 3D single-molecule experiments.*

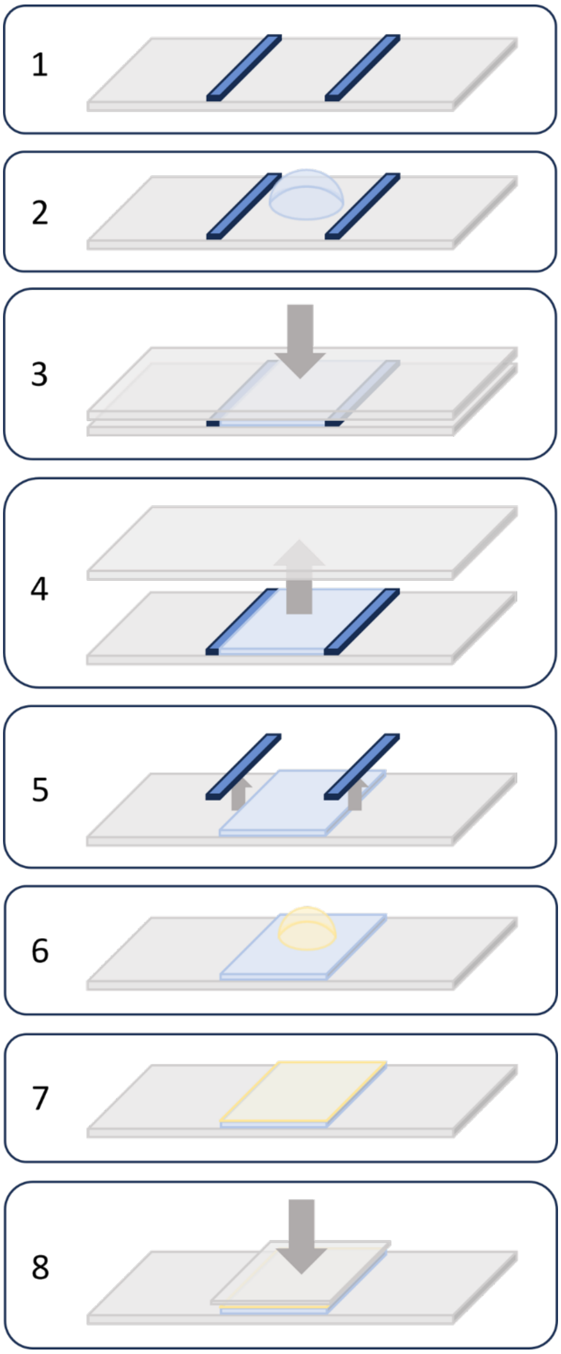

1. Take a microscope slide and use thin strips of scotch tape (about 300 µm) to create a square region of around 24x24 mm^2.

2. Drop 200 µl of liquid 2% agarose in the center of the square. If the agarose is prepared with microwave, wait 5-10 s before the next step. If the agarose temperature is too high when it's covered, it will come out of the region and the pad won't form properly.

3. Cover with another microscope slide and hold it down until the agarose solidifies. Be careful not to create bubbles during the covering because they can create issues during the imaging process.

4. Carefully remove the upper microscope slide.

5. Carefully remove the thin strips of scotch tape.

6. Take 20 µl of bacteria suspended in PBS and drop it on the surface of the solidified agarose pad.

7. Wait until it dries and the bacteria are completely embedded with the pad (~30 minutes).

8. Cover with a 24x24 mm^2 coverslip. If the agarose is too dry, put few µl of PBS to ease the attachment of the coverslip.

Figure S3: *Step by step assembly protocol of the agarose pad with embedded bacteria used for 2D PALM acquisitions.*

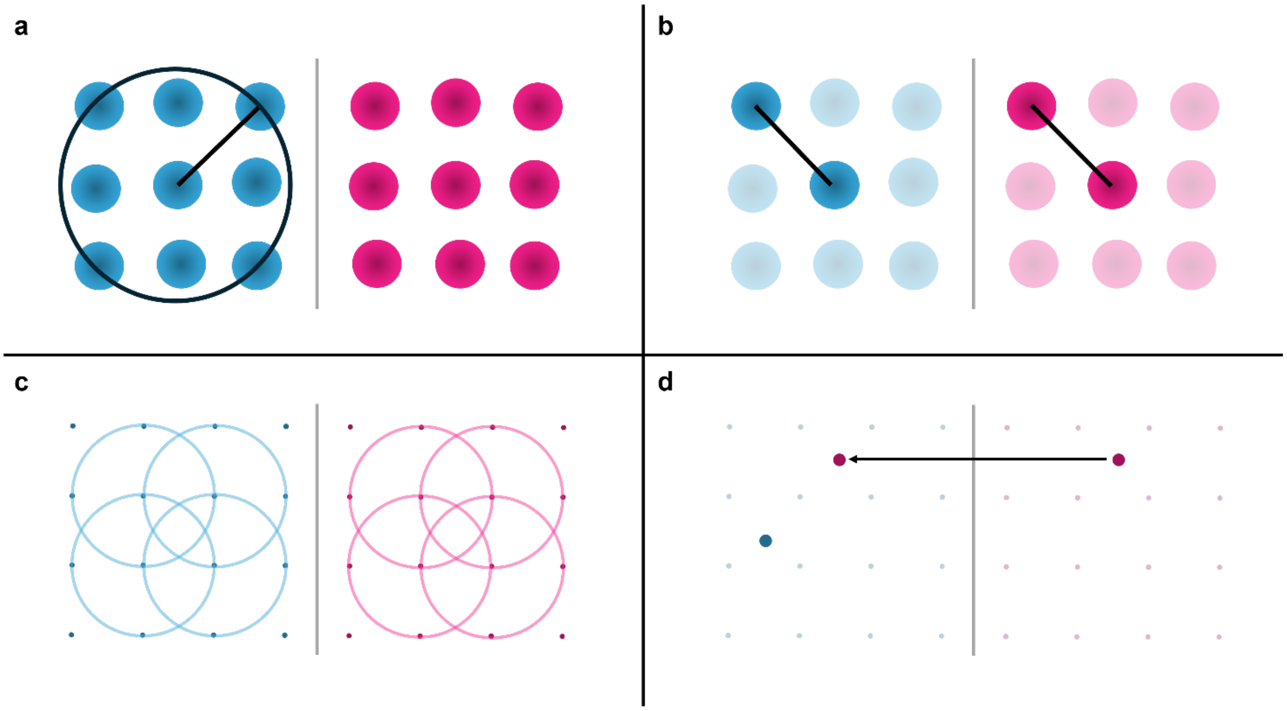

Figure S4: *Main steps of the algorithm used to correct for chromatic aberrations. a) The algorithm selects a control point and the corresponding control point in the other channel. Then it measures the distance between the control point and the 8 nearest adjacent points; b) the longer distance is selected as the radius of influence of the transformation function that will be calculated for that point and, therefore, for that area; c) the algorithm infers a polynomial at each control point using neighbouring control points. This results in a local transformation function and an associated radius of influence for it. By repeating the same series of calculations for each control point, we end up with a series of polynomial transformation functions and their associated radii of influence; d) The global transformation function at an arbitrary point is calculated as weighted sum of polynomials having a nonzero weight over that point. Thanks to the transformation function, we can map each point of the field of view to the corresponding coordinate in the other channel. Once an object in the red channel is localized, its position relative to the control points is used to calculate the corresponding position in the green channel.*

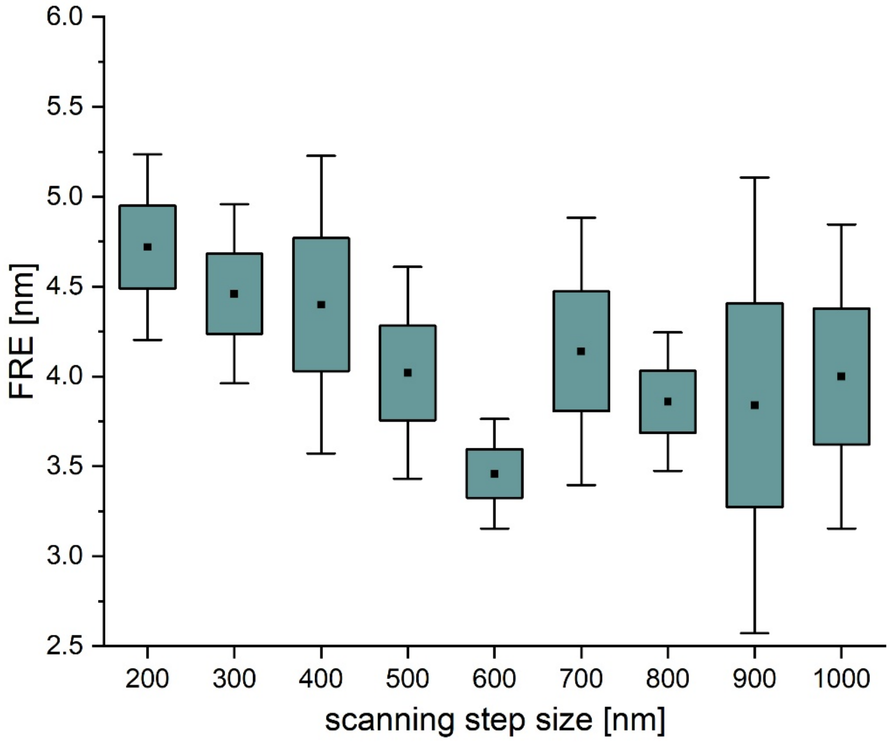

Figure S5: *Comparison of FRE between different scanning step sizes. In each box plot, the square represents the mean value, the box is ± 1 SE and the whiskers are ± 1 SD.*

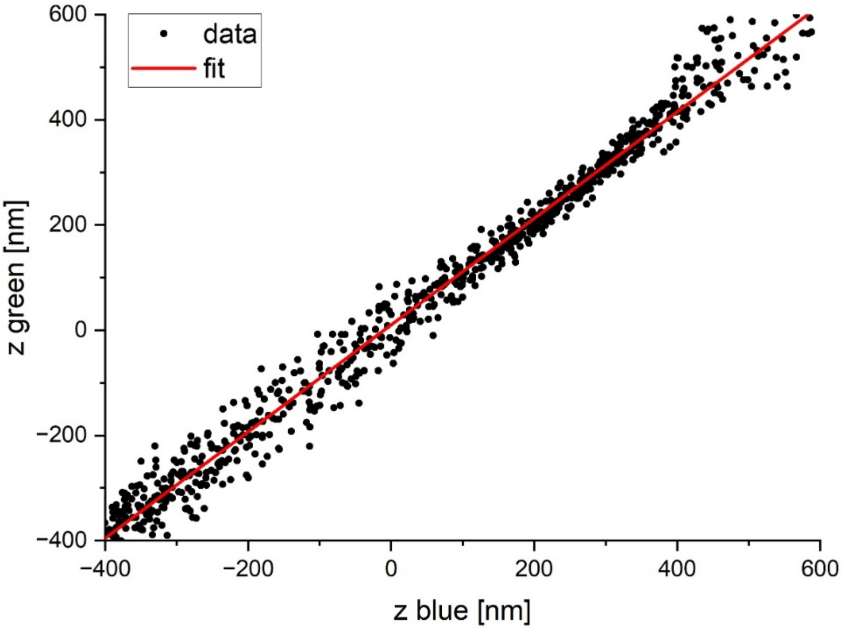

Figure S6: *Estimate of the focal shift between the two channels due to axial chromatic aberration. The dark spots represent the z coordinate in the blue (abscissa axis) and green (ordinate axis) channels while the red line is the linear fit. The results of the fit are intercept = (9.8 ± 1.3) nm and slope = 1.013 ± 0.005.*

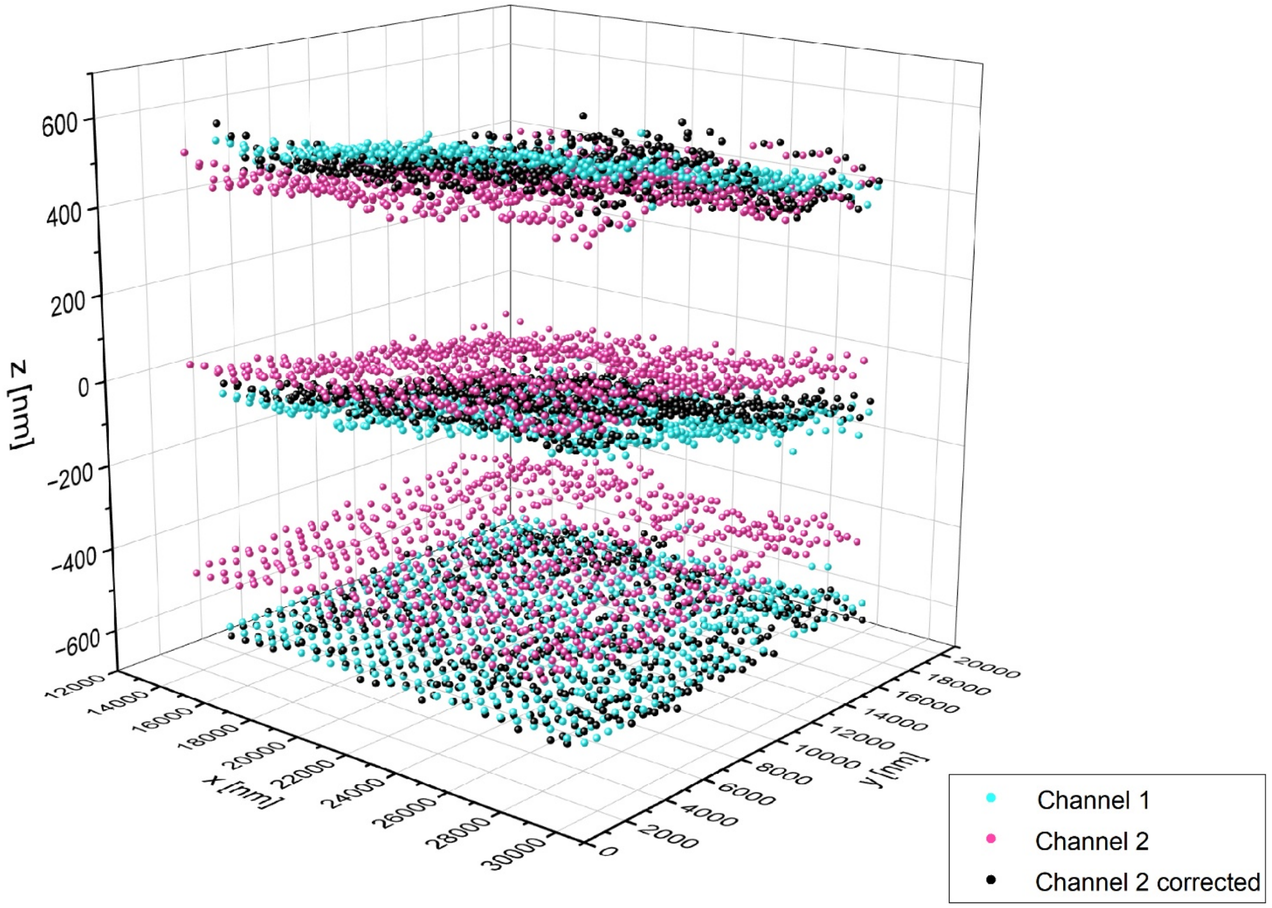

Figure S7: *Example of the calibration grid used to estimate the transformation function and correct chromatic aberration in 3D. The correction is performed over the axial range ±600 nm. From these calibration points, a 3D transformation function is derived to correct chromatic aberration yielding a $FRE_{x,y,z}$ of 6.4 nm (calculated using Equation 1 of the article), after rescaling by the expansion factor. The total FRE is 11 nm, obtained as quadratic sum of the registration errors along the three axes.*

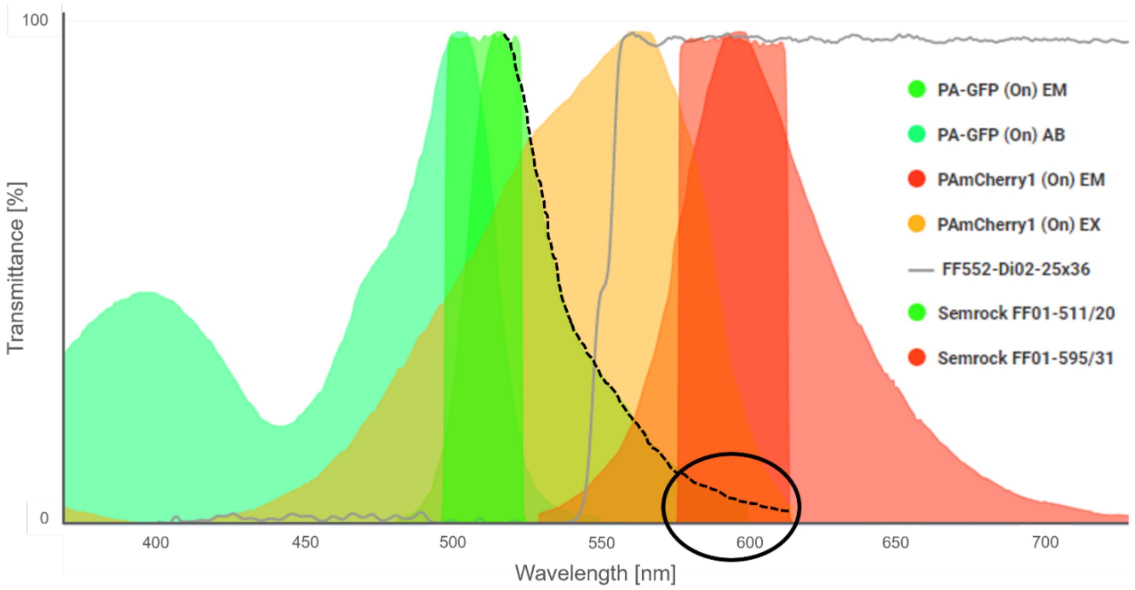

Figure S8: *Transmittance as a function of wavelength of fluorescent proteins (PAGFP and PAmCherry1) and detection optical elements (dichroic mirrors and fluorescent filters) of our setup. The dashed line delimits the right part of the emission spectrum of PAGFP while the black circle highlights the crosstalk signal of PAGFP in the PAmCherry1 channel. Spectra retrieved from the online dataset FPbase (https://www.fpbase.org/spectra/).*

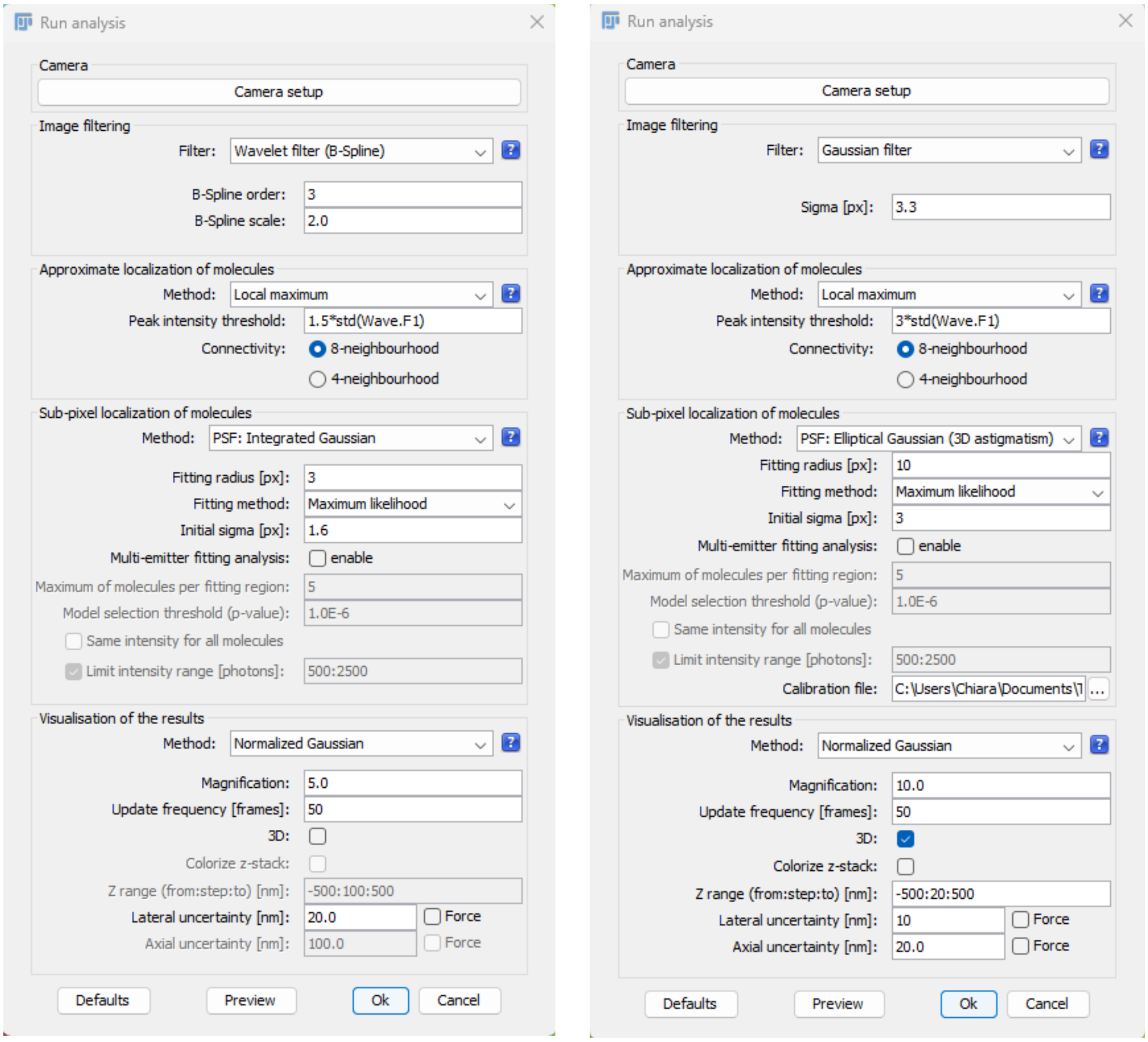

Figure S9: *ThunderSTORM user interface showing the parameters used to find the sub-pixel positions of single molecules of PAGFP and PAmCherry1 in bacteria in simulations, 2D PALM and 2D Ex-PALM (left) and 3D Ex-PALM (right)*[5].

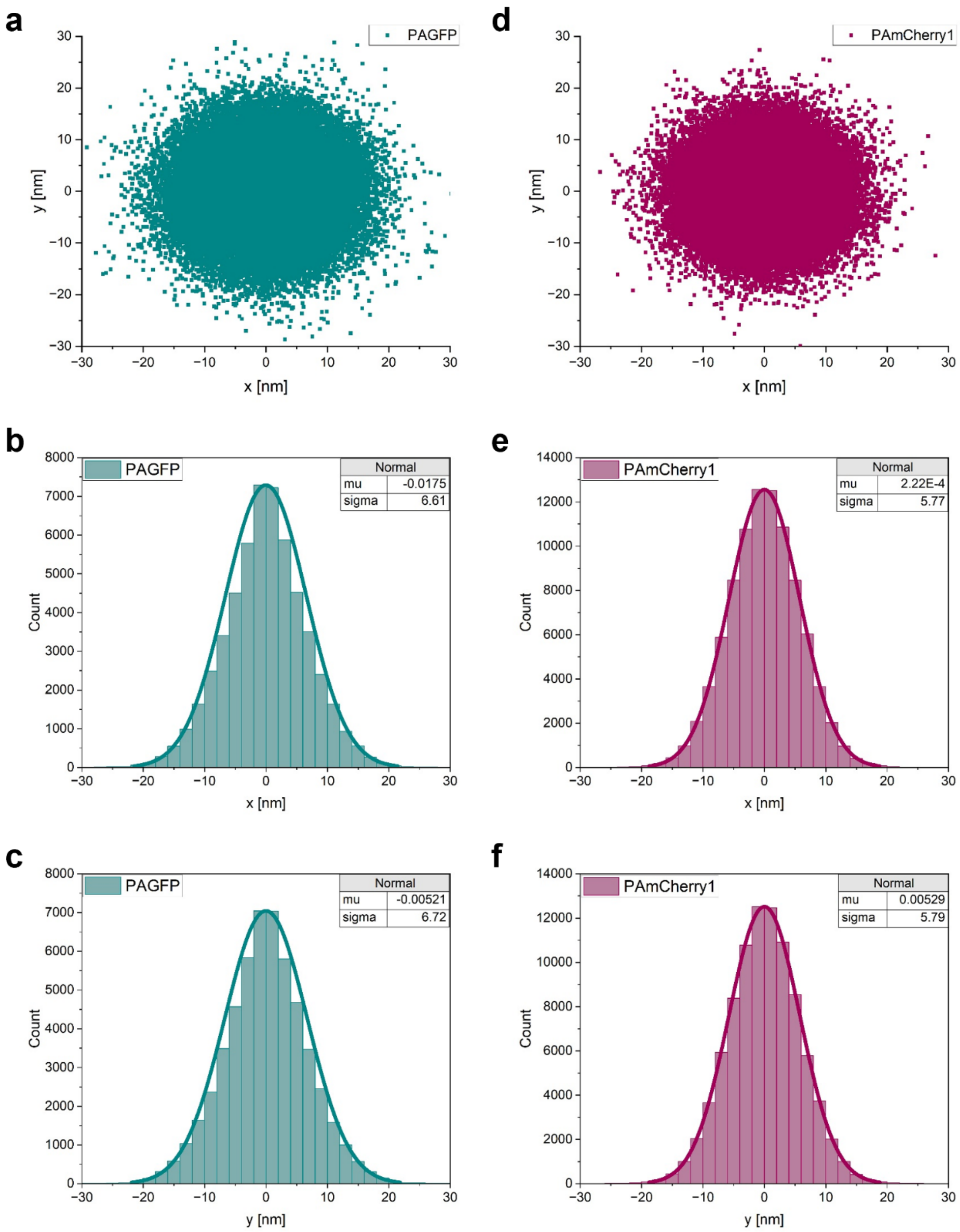

Figure S10: *Localization precision of PAGFP (cyan, N = 25122) and PAmCherry1 (magenta, N = 39505), estimated from repeated localizations of the same molecules obtained from ten 2D PALM acquisitions. (a, d) Two-dimensional localization distributions of single molecules. (b, c, e, f) Histograms of the localization distributions along the two axes, fitted with Gaussian functions. The centroid and the standard deviation of each fit are reported in the top right corner of each histogram.*

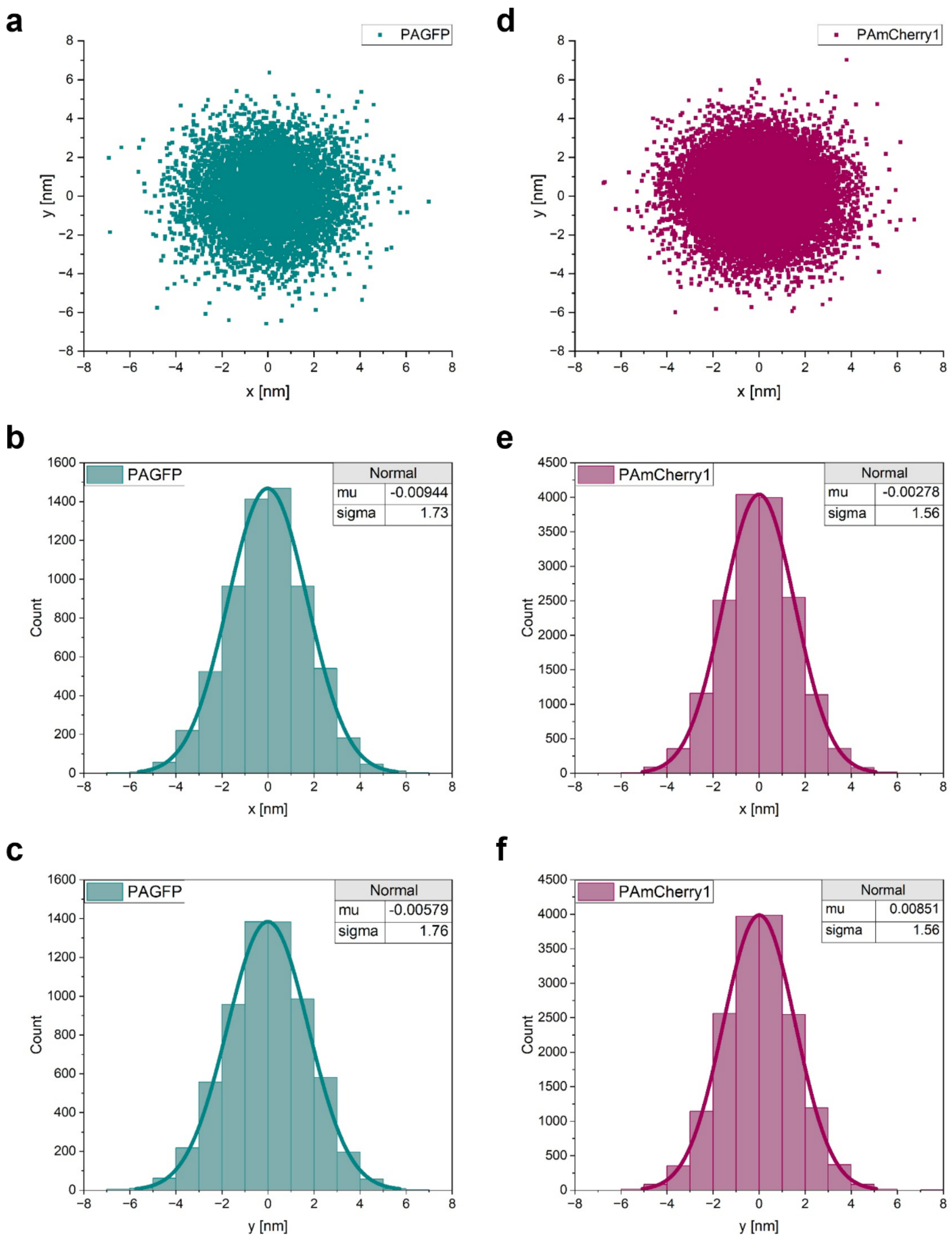

Figure S11: *Localization precision of PAGFP (cyan, N = 2767) and PAmCherry1 (magenta, N=6876), estimated from repeated localizations of the same molecules obtained from ten 2D Ex-PALM acquisitions. (a, d) Two-dimensional localization distributions of single molecules. (b, c, e, f) Histograms of the localization distributions along the two axes, fitted with Gaussian functions. The centroid and the standard deviation of each fit are reported in the top right corner of each histogram.*

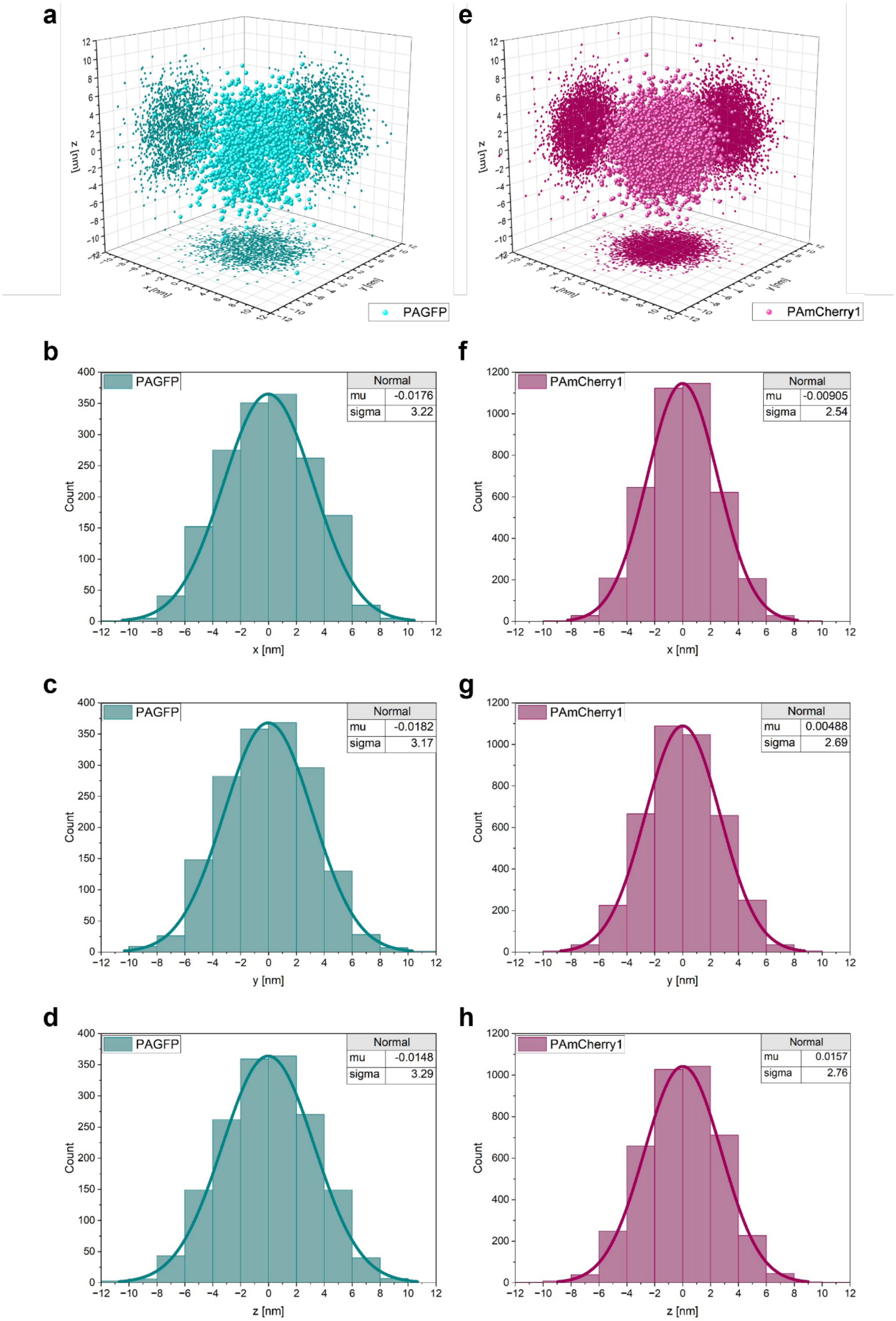

Figure S12: *Localization precision of PAGFP (cyan, N=778) and PAmCherry1 (magenta, N=2173) , estimated from repeated localizations of the same molecules obtained from ten 3D Ex-PALM acquisitions. (a, e) Three-dimensional localization distributions of single molecules. (b-d, f–h) Histograms of the localization distributions along the three axes, fitted with Gaussian functions. The centroid and the standard deviation of each fit are reported in the top right corner of each histogram.*

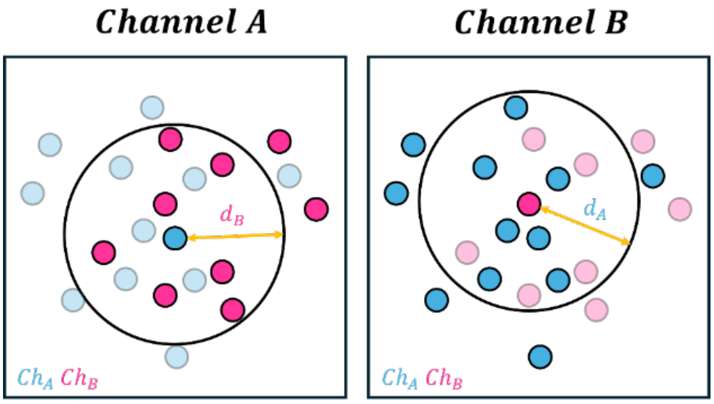

$$CI_i^A = \frac{N_{Ai}^B(d_B)}{(\overline{LD}_B)} \qquad CI_i^B = \frac{N_{Bi}^A(d_A)}{(\overline{LD}_A)}$$

Figure S13: *Concept of local density-based co-localization index. The co-localization index for each localization of channel A can be defined as the number of localizations in channel B (N_{Ai}^B) within a radius (d) around the i-th localization in channel A normalized to the mean local density of the localizations in channel B ($\overline{LD}_B$). The radius d is the effective resolution of the system, calculated as the quadratic sum of the localization error and the mean nearest-neighbour distance*[6].

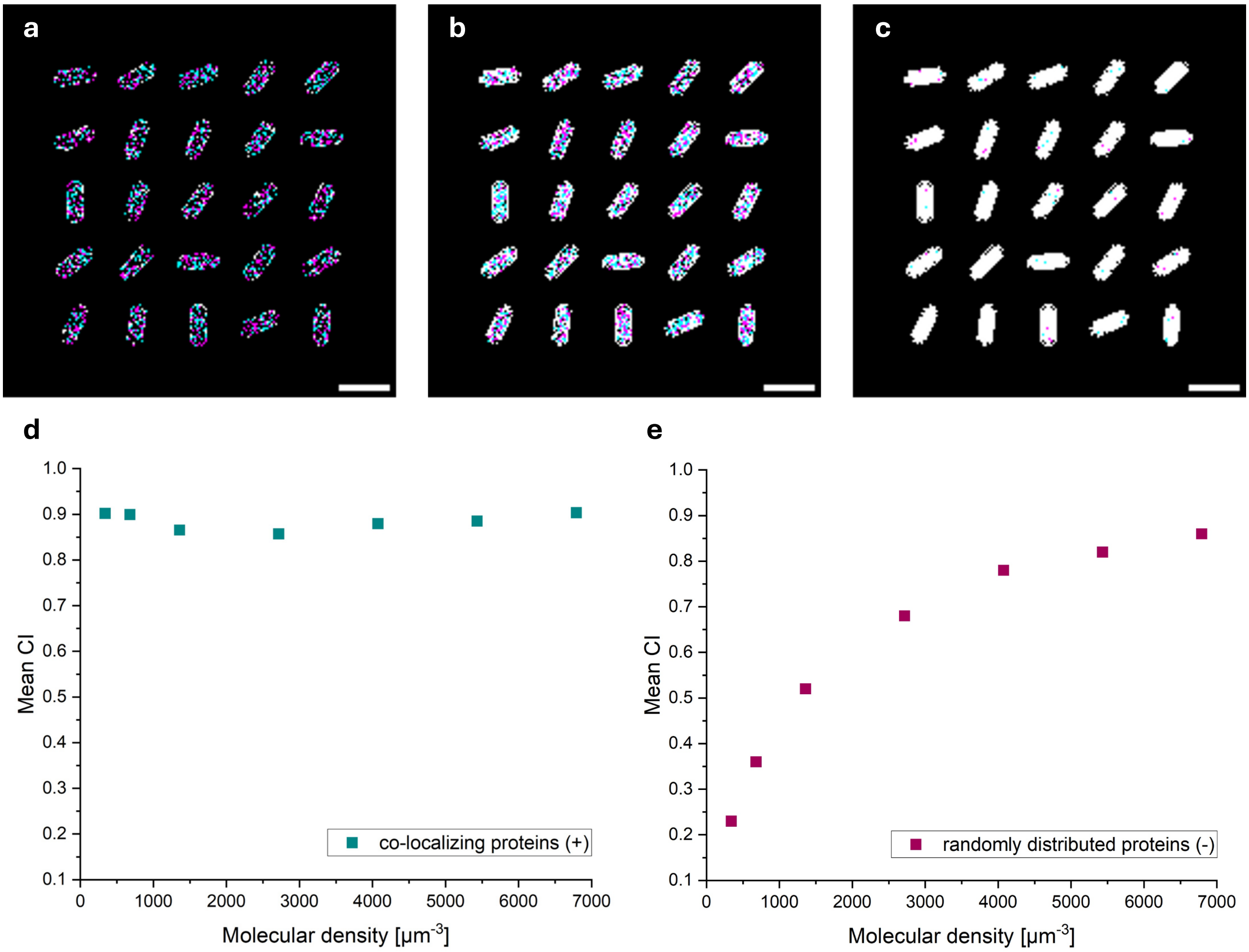

Figure S14: *Relation between molecular density of fluorophores and co-localization analysis. (a, b, c) Simulations of randomly distributed PAGFP (cyan) and PAmCherry1 (magenta) in E. coli bacteria with increasing molecular density (from 340 to 6800 molecules/μm^3); (d, e) Mean CIs with respect to the average molecular density for co-localizing (cyan, left) and randomly distributed (magenta, right) fluorescent proteins. The higher is the molecular density of the fluorophores, the harder it becomes to distinguish the random distributions from the co-localizing ones. Scalebar: 2 µm. Simulations performed using the program SMIS*[7].

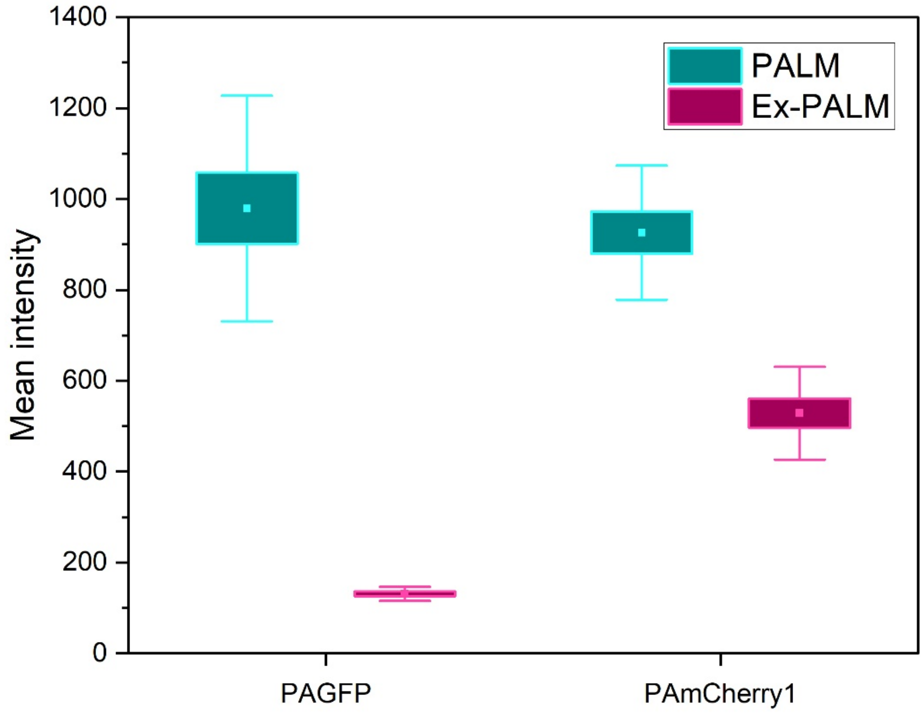

Figure S15: *Protein fluorescence signal retention after expansion. Box plots comparing the mean fluorescence intensity of PAGFP and PAmCherry measured in PALM (N = 10) and Ex-PALM (N = 10) acquisitions. In each box plot, the square represents the mean value, the box is ± 1 SE and the whiskers are ± 1 SD. Relative to PALM, PAGFP is more strongly affected by the expansion protocol, retaining only ~13% of its fluorescence signal, whereas PAmCherry retains ~57%.*

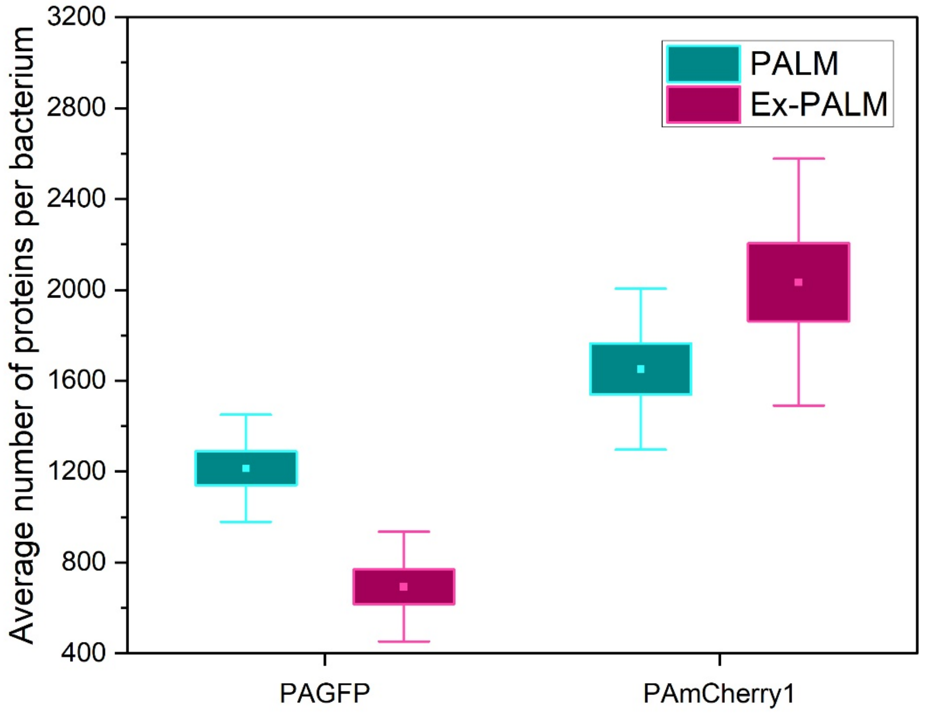

Figure S16: *Protein retention after expansion. Box plots comparing the average number of PAGFP and PAmCherry localizations per bacterium measured in PALM (N = 10) and Ex-PALM (N = 10) acquisitions. In each box plot, the square represents the mean value, the box is ± 1 SE and the whiskers are ± 1 SD. Relative to PALM, PAGFP is more strongly affected by the expansion protocol, retaining ~57% of its signal, whereas PAmCherry shows an apparent ~23% increase in localizations. This apparent increase does not indicate improved retention but arises from the higher effective resolution in Ex-PALM, which reduces merging of closely spaced molecules during duplicate removal and thus decreases undercounting compared to conventional PALM.*

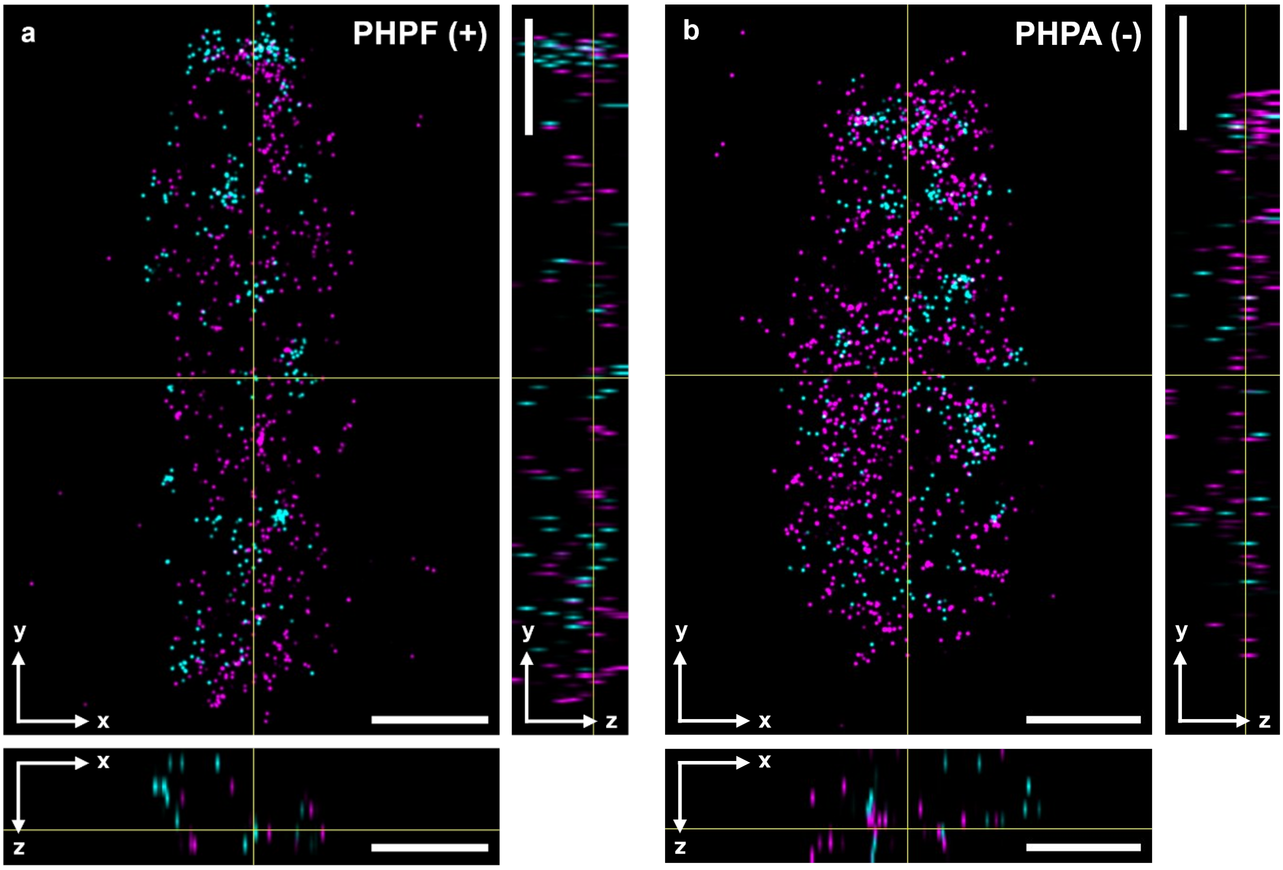

Figure S17: *Orthogonal projections of the 3D Ex-PALM acquisitions shown in Figure 7 of the manuscript. (a) PHPF sample and (b) PHPA sample. PAGFP localizations are shown in cyan and PAmCherry1 localizations in magenta. Axial range: ±500 nm. Scalebar: 1 µm. The images shown are rotated by 30° (for PHPF) and 80° (for PHPA) with respect to the original data in order to perform the orthogonal projections.*

Supplementary Tables

	Simulations			Acquisitions		
	Co-localization Index (CI)	Percentage of co-localizing molecules	Mean Distance [nm]	Co-localization Index (CI)	Percentage of co-localizing molecules	Mean Distance [nm]
**PHPF (+)**	0.940 ± 0.002	0.9157 ± 0.0006	16.37 ± 0.06	0.905 ± 0.006	0.822 ± 0.005	20.2 ± 0.6
**PHPA (-)**	0.893 ± 0.002	0.9044 ± 0.0006	16.98 ± 0.06	0.884 ± 0.004	0.797 ± 0.004	23 ± 3

Supplementary Table S1: *Results of the co-localization analysis of multicolour PALM simulations and acquisitions. For each parameter is reported the mean value and its standard error.*

	2D Ex-PALM			3D Ex-PALM		
	Co-localization Index (CI)	Percentage of co-localizing molecules	Mean Distance [nm]	Co-localization Index (CI)	Percentage of co-localizing molecules	Mean Distance [nm]
**PHPF (+)**	0.50 ± 0.03	0.419 ± 0.017	19.1 ± 1.0	0.47 ± 0.03	0.400 ± 0.017	19.6 ± 1.0
**PHPA (-)**	0.39 ± 0.04	0.34 ± 0.02	26.4 ± 1.7	0.37 ± 0.04	0.34 ± 0.03	25 ± 3

Supplementary Table S2: *Results of the co-localization analysis of multicolour 2D and 3D Ex-PALM imaging. For each parameter is reported the mean value and its standard error.*

Supplementary Protocols

Scanning of the field of view to correct for chromatic aberrations

To perform the scanning of the field of view a sample with multicolour fluorescent beads is required. To prepare the sample, we use a 26×76 mm² microscope slide, a 24×40 mm² coverslip, double-sided tape of approximately 100 µm thickness, TetraSpeck™ microspheres (ThermoFisher, T7279, 0.1 µm, blue/green/orange/dark red), and phosphate-buffered saline (PBS) 1×. A two-channel chamber is constructed using double-sided tape, as shown in **Figure S18**. Although only one channel is necessary for each solution, using two allows measurements under different conditions within the same chamber. The microspheres are diluted 1:1000 in PBS, and 20 µl of this dilution is placed into one channel and left for 15 minutes. The sample is then washed with 40 µl of PBS. This prepared sample remains usable for several hours. For extended use over several days, sealing with silicone grease is essential to prevent drying.

Once the sample is prepared, the scanning of the field of view is performed as follows. The camera gain is set to 200, and the exposure time is set to 100 ms. The grid size and scanning step size are selected, with all parameters for different step sizes detailed in **Supplementary Table S3**. The total acquisition time depends not only on the number of frames and exposure

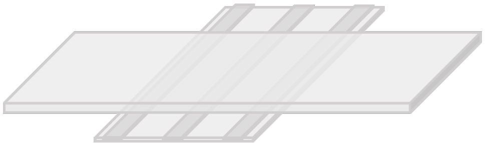

Figure S18: *Two-channel chamber composed of a rectangular coverslip attached on a microscope slide through thin strips of double-sided tape, which also delimit the channels. Each channel has a volume of 20 µl. Solutions are fluxed into the channels by gently pipetting through one of the open ends.*

Step size [nm]	Columns	Rows	Images per step	Number of frames	Acquisition time [min]
100	380	160	1	60800	182.3
200	190	80	1	15200	45.6
300	127	53	1	6731	20.2
400	95	40	1	3800	11.4
500	76	32	1	2432	7.3
600	63	27	1	1701	5.1
700	54	23	1	1242	3.7
800	48	20	1	960	2.9
900	42	18	1	756	2.3
1000	38	16	1	608	1.8

Supplementary Table S3: *Scanning parameters for different scanning step sizes covering a field of view of around 38 x 16 µm^2.*

time but also on the scanning procedure itself, as approximately 80 ms is required to turn off the camera, reposition the stage, and reactivate the camera. After setting these parameters, the 552 nm laser is turned on at 0.8 mW (~34 W/cm² out of the objective), and the sample is scanned to find a region containing one or a few isolated beads. The algorithm used for transformation function determination identifies the reference bead and discards others if they are farther than 100 nm. However, if multiple beads occupy the same position at different times, their localizations may appear closer than 100 nm when reconstructing the grid image. This could cause the algorithm to select an incorrect localization, compromising the accuracy of the transformation function. Once a suitable region of interest is selected, the 488 nm laser is activated at the same power as the 552 nm laser, and the acquisition begins. An automated customed LabVIEW program sequentially captures images, turns off the camera, moves the stage to the next position, and reactivates the camera. After all frames have been recorded, the camera is stopped, and the lasers are turned off.

To optimize the scanning step size, calibrations were performed using step sizes from 200 nm to 1000 nm. Since the calibration grid must cover most of the field of view for effective correction, but fluorescent bead photobleaching over time affects localization precision, a balance between grid coverage and transformation precision is required. To find the best scanning step size (i.e. the one with the lowest FRE), we performed five acquisitions for each step size and then estimated the FRE for each. A comparison of the registration errors of different acquisitions is reported in **Figure S5**. The lowest mean FRE was observed at 600 nm.

PALM and Ex-PALM sample preparation protocol

Materials

- *E. coli* bacteria glycerol stock
- Culture medium: M9 minimal medium with added glucose (0.4%), histidine (25 µg/ml), IPTG (50 µg/ml) and kanamycin (50 µg/ml)
- 4% Paraformaldehyde (PFA)
- Phosphate-buffered saline (PBS) 1x
- PBSTx: 0.3% (w/v) Triton X-100 in PBS
- 50% Methanol in PBSTx
- TAE buffer: 40 mM Tris base, 20 mM Acetic acid and 1 mM Ethylenediaminetetraacetic acid (EDTA) in MilliQ H_2O
- Methacrylic Acid N-HydroxySuccinimide ester (MA-NHS) stock solution: 1 M MA-NHS in dimethyl sulfoxide (DMSO)
- Phosphate buffer: 50 mM potassium phosphate in MilliQ H_2O. Adjust pH to 4.9. This solution should be made fresh before use.

- Mutanolysin stock solution: mutanolysin 10000 u/ml in MilliQ H_2O.
- Monomer solution: 2 M NaCl, 2.5% (w/w) acrylamide, 0.15% (w/w) bisacrylamide, 8.625 % (w/w) sodium acrylate in PBS.
- Gelation solution: 0.01% (v/v) 4-hydroxy-TEMPO, 0.2% (w/v) ammonium persulfate (APS), and 0.2% (v/v) tetramethylethylenediamine (TEMED) in monomer solution. This solution should be made fresh in ice just before the gelation step. APS should be added at last.
- Digestion buffer: 0.5% Triton X-100, 0.8 M guanidine HCl and 8 units/ml Proteinase K (ProK) in TAE buffer. The digestion buffer, without ProK, must have its pH adjusted to 8.0, as ProK is highly dependent on pH and temperature. This solution can be stored at -20°C for several months. ProK should be added just before use.
- 0.1% Poly-L-lysine solution
- 13-mm, 18-mm and 25-mm diameter coverslips
- 100-mm petri dishes
- 6-well plate

Methods

1) Cell growth and fixation (same for PALM and Ex-PALM samples)

- Grow bacteria in 5 ml of culture medium for about 31 hours at 37°C and 215 rpm.
- Collect cells via centrifugation at 10000g for 2 minutes and resuspend the pellet in 1 ml of PBS.
- Wash once with PBS via repeated centrifugation and resuspension, then incubate in PFA 4% for 10 minutes.

2a) PALM protocol

- Replace the fixative with PBS and follow the procedure shown in **Figure S3**

2b) Ex-PALM protocol

- Replace the fixative with PBS and measure the optical density at 600 nm (OD_{600}). It should be between 1.8-2.1. This measurement is crucial because the amount of mutanolysin used for cell wall digestion depends on the bacterial concentration, and the protocol is optimized for this OD range. If the optical density is higher than this range, the standard amount of mutanolysin may be insufficient for complete digestion, requiring a higher concentration. On the other hand, if the optical density is lower, the mutanolysin concentration may be excessive and could damage the bacteria.
- Remove PBS and resuspend in 1 ml of PBSTx by gentle pipetting. Incubate cells for 30 minutes at room temperature on a revolver rotator mixer to permeabilize the membrane.

- Centrifuge at 2000g for 5 minutes and resuspend in 1 ml of 50% methanol in PBSTx and incubated with agitation for 10 minutes. Since bacteria become more fragile after permeabilization, all subsequent centrifugation steps are performed at 2000g to prevent damage.
- Remove the solution and incubate in PBSTx for 10 minutes with agitation.
- Remove PBSTx and digest cell walls by incubating in 1 ml of phosphate buffer containing 320 unit/ml mutanolysin overnight at 37 °C on a rotating mixer.
- Wash in PBS three times and then incubate in 1 ml of 2 mM MA-NHS in PBS for 1 hour at room temperature to chemically anchor proteins.
- Wash in PBS three times, then replace PBS with 78 µl gelation solution and incubate for 1 minute at 4 °C. Each sample (bacteria pellet diluted in the gelation solution) can yield three gels. Since gels are fragile and could easily break or get ruined in the further steps for several different reasons it is better to have some spare ones. In this step, be careful to remove all the PBS before resuspending the pellet with the gelation solution. If some PBS is left, the gel won't form properly, and it will break in next steps or it will expand non-isotropically.
- To ensure successful gelation, a humid gelation chamber is used to prevent drying. This is assembled by placing a moist piece of paper in each well of a 6-well plate, positioning an 25-mm diameter coverslip at the centre of each well, and adding 26 µl of bacterial suspension to the coverslip's centre (as shown in **Figure S19**). A plasmed 13-mm diameter coverslip is carefully placed over the droplet, and the process is repeated for all wells. The plate is then incubated for 2 hours at 37°C, during which the cells settle primarily near the surface in contact with the 18-mm diameter coverslip.

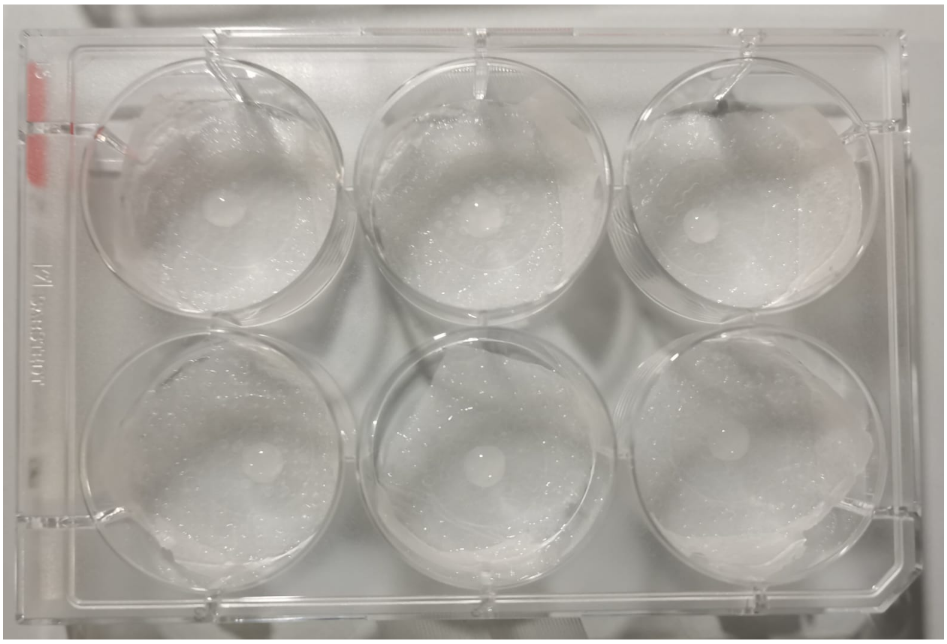

Figure S19: *Assembling of the gelation chamber. Each well contains a piece of humid paper surrounded by an 18-mm diameter coverslip with 26 µl of bacterial suspension. To finalize the chamber, put a 13-mm diameter coverslip on the top.*

- Carefully remove the upper coverslip from the gel and the humid paper from each well. After repositioning the gel in its well, add 2.5 ml of digestion buffer with proteinase K, seal the multi-well with parafilm to prevent evaporation and incubate for 3 hours at 37 °C. During digestion, the gel expands by a factor of approximately 1.5, making it more suitable to use a 6-well plate instead of a 12-well plate to accommodate the increased gel size.
- Transfer the gel into a 100-mm petri dish using the lower coverslip, a needle and tweezers to facilitate the movement. Once the gel is in position, fill the dish with MilliQ H_2O and allow the gel to expand at room temperature for 45 minutes, changing the water every 15 minutes. Alternatively, expansion can be performed overnight at 4 °C.
- For sample mounting, coat the coverslip of the imaging chamber with 0.1% poly-L-lysine for 5 minutes.
- Remove the expansion water from the gel and cut it into small pieces to be imaged. Finally, take a piece of gel and gently place it into the imaging chamber with some expansion water to prevent drying of the gel.

Once prepared, the sample in the imaging chamber can be used for several hours.

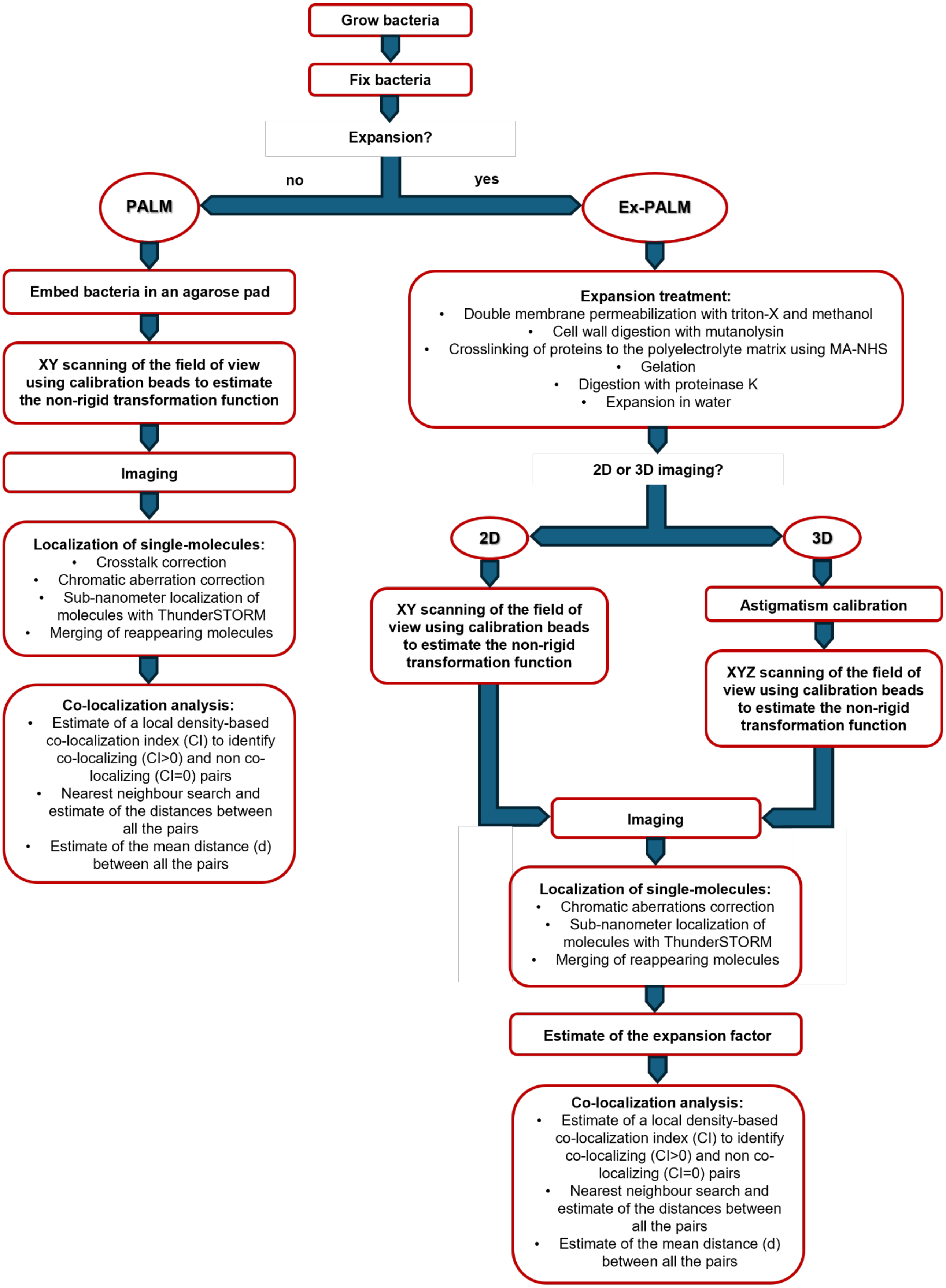

Figure S20: *Flowchart summarizing the experimental workflow from sample preparation to co-localization analysis.*

Bibliography

1. Gardini, L., Vignolini, T., Curcio, V., Pavone, F.S., and Capitanio, M. (2023). Optimization of highly inclined illumination for diffraction-limited and super-resolution microscopy. Optics Express, Vol. 31, Issue 16, pp. 26208-26225 *31*, 26208–26225. https://doi.org/10.1364/OE.492152.

2. Vignolini, T., Capitanio, M., Caldini, C., Gardini, L., and Pavone, F.S. (2024). Highly inclined light sheet allows volumetric super-resolution imaging of efflux pumps distribution in bacterial biofilms. Sci Rep *14*. https://doi.org/10.1038/S41598-024-63729-X.

3. Huang, B., Wang, W., Bates, M., and Zhuang, X. (2008). Three-dimensional super-resolution imaging by stochastic optical reconstruction microscopy. Science (1979) *319*, 810–813. https://doi.org/10.1126/science.1153529.

4. Kashchuk, A. V, Perederiy, O., Caldini, C., Gardini, L., Pavone, F.S., Negriyko, A.M., and Capitanio, M. (2022). Particle Localization Using Local Gradients and Its Application to Nanometer Stabilization of a Microscope. https://doi.org/10.1021/acsnano.2c09787.

5. ThunderSTORM: a comprehensive ImageJ plug-in for PALM and STORM data analysis and super-resolution imaging | Bioinformatics | Oxford Academic https://academic.oup.com/bioinformatics/article/30/16/2389/2748167.

6. Willems, J., and MacGillavry, H.D. (2022). A coordinate-based co-localization index to quantify and visualize spatial associations in single-molecule localization microscopy. Scientific Reports 2022 12:1 *12*, 1–12. https://doi.org/10.1038/s41598-022-08746-4.

7. Bourgeois, D. (2023). Single molecule imaging simulations with advanced fluorophore photophysics. Communications Biology 2023 6:1 *6*, 1–13. https://doi.org/10.1038/s42003-023-04432-x.
